# Sleep-Related Breathing Disorders: A Comprehensive Review of Surgical Innovations and Evolving Technologies

**DOI:** 10.3390/healthcare14142069

**Published:** 2026-07-10

**Authors:** Amrit Kooner, Lee Man, Justin Best, Nicholas Litsky, Brianna Yee, Justin Jeffries

**Affiliations:** 1 Department of Internal Medicine, Kirk Kerkorian School of Medicine at UNLV, Las Vegas, NV 89106, USA; lee.man@unlv.edu (L.M.); justin.best@unlv.edu (J.B.); nicholas.litsky@unlv.edu (N.L.); 2 Department of Cardiology, Icahn School of Medicine at Mount Sinai, New York, NY 10029, USA; brianna.yee@mountsinai.org; 3 Department of Pulmonary and Critical Care, Kirk Kerkorian School of Medicine at UNLV, Las Vegas, NV 89106, USA; justin.jeffries@unlv.edu

**Keywords:** sleep-related breathing disorders, obstructive sleep apnea, sleep surgery, hypoglossal nerve stimulation, drug-induced sleep endoscopy, precision medicine, artificial intelligence, maxillomandibular advancement, management and diagnosis

## Abstract

Sleep-related breathing disorders (SRBDs) encompasses a spectrum of conditions that disrupt ventilation during sleep, leading to fragmented sleep and impaired gas exchange. Their high prevalence and substantial neurocognitive and mental health outcomes make SRBD clinically significant across multiple medical disciplines. Traditional management includes lifestyle modifications and positive airway pressure (PAP). When non-surgical measures fail or anatomical factors predominate, a range of surgical approaches may be employed, such as uvulopalatopharyngoplasty (UPPP) or maxillomandibular advancement (MMA). There are many notable emerging surgical advancements, such as hypoglossal nerve stimulation (HNS), transoral robotic surgery (TORS), and minimally invasive radiofrequency technologies (RFA), that have offered improved outcomes for select patients. Advances in diagnostic tools, such as portable home sleep technologies and drug-induced sleep endoscopy (DISE), further support precision-based care. Collectively, the expanding range of therapeutic and diagnostic innovations is enabling clinicians to deliver individualized care and improve long-term outcomes for patients with SRBD.

## 1. Introduction

### 1.1. Overview of Sleep-Related Breathing Disorders (SRBDs)

Sleep-related breathing disorders (SRBDs) are a broad group of conditions that result in abnormal breathing during sleep [1]. These conditions disrupt gas exchange, leading to hypercapnia, surges in sympathetic tone, and fragmentation of the sleep cycle [2]. Several anatomic features can correlate with the development of SRBD, including micrognathia, excessive soft tissue around the neck, and a narrow oropharynx [3]. General symptoms often include snoring, frequent nighttime awakenings, daytime sleepiness, impaired memory, increased risk of motor collisions, and mental health issues, including depression.

The primary categories of SRBD include obstructive sleep apnea (OSA), central sleep apnea (CSA), sleep-related hypoventilation disorder, and sleep-related hypoxemia disorders [4]. Obstructive hypopnea refers to a partial reduction in airflow, whereas obstructive apnea involves a complete obstruction of the upper airway or pharynx. In contrast, CSA is a result of the transient inhibition of the brainstem response rather than an anatomic obstruction. Presenting symptoms include frequent arousals through the night, loud snoring, and even episodes of desaturation [5]. Risk factors for the development of SRBD include male gender, obesity, head trauma, and tonsillar hypertrophy, as well as craniofacial variations such as retrognathism, micrognathism, or macroglossia [5]. Obesity is a significant risk factor due to fat deposition in the parapharyngeal space, which can compress the upper airway.

### 1.2. Significance of SRBD in Clinical Practice

Sleep plays a critical role in nearly every organ system, with a significant impact on overall quality of life. The prevalence of SRBD is approximately 32.4% in the United States in adults over the age of 20, which makes this a highly prevalent disease [6]. Long-term consequences of SRBD include a range of cardiometabolic effects such as hypertension, chronic kidney disease, heart failure, and pulmonary hypertension [7]. SRBDs are closely associated with obesity, which contributes to systemic inflammation and oxidative stress from reactive oxygen species (ROS). These mechanisms promote endothelial injury through chronic macrophage activation and heightened sympathetic activity, in turn increasing the risk of atherosclerotic disease [8].

### 1.3. Purpose of This Review

This review aims to provide an overview of the evolving technologies used to treat SRBD and a comprehensive review of current treatment modalities. Accordingly, this paper will predominantly focus on OSA, the most extensively studied form of SRBD, with the clearest evidence base and well-defined indications for available interventions. However, where relevant, overlap with other forms of SRBD, particularly CSA and mixed sleep apnea phenotypes, will be discussed to provide broader pathophysiologic and therapeutic context. Current therapies range from lifestyle modifications to supportive devices such as positive airway pressure (PAP) therapy, oral appliances, and surgical techniques designed to address anatomic concerns to keep the airway patent. The surgical section will outline established interventions such as maxillomandibular advancement and uvulopalatopharyngoplasty, and the modifications that have continued to evolve.

We aim to address the expansion of SRBD with new technologies such as implantable devices for nerve stimulation and the incorporation of virtual surgical planning with computer-aided design. Although the gold standard of diagnosis remains polysomnography, this paper will also highlight new diagnostic testing in the home setting with emerging technologies. As the understanding of genetic, anatomic, and molecular factors related to SRBD continues to grow, this manuscript will address the significance and role of developing a personalized approach for each patient.

## 2. Methods

The PubMed, Cochrane, and Google Scholar databases were used for the literature review. The primary research question was “What are the pathophysiologic factors of SRBD and what are existing treatments?” Keywords for this search included SRBD, OSA, sleep-disordered breathing, UPPP, MMA, hypoglossal nerve stimulation, radiofrequency ablation, and minimally invasive surgeries. Initially, the search was limited to studies published from 2015 onward; this was expanded to include landmark historic data to provide a comprehensive review of this topic. Preference was given to randomized controlled trials, systematic reviews, meta-analyses, observational studies, epidemiological studies, and retrospective studies. Because this paper focuses on the treatment of adults, studies involving pediatric populations were omitted.

## 3. Growing Overlap Within SRBD

Although OSA remains the primary focus of most surgical interventions in SRBD, growing evidence highlights that the distinction between OSA and CSA may not always be absolute. In contrast to OSA, which is characterized by upper airway collapse, CSA is attributed to instability of respiratory drive and ventilatory control [9]. However, many contemporary models increasingly recognize that patients may have overlapping physiologic traits, including altered arousal threshold, ventilatory instability, and upper airway dysfunction. As a result, SRBD is increasingly viewed as a spectrum of pathophysiologic mechanisms rather than completely separate diseases.

This framework is relevant in patients with mixed or complex sleep phenotypes. Effective treatment of upper airway obstruction may influence ventilatory control through several indirect mechanisms. Particularly, patients with mixed sleep apnea phenotypes who may have resolution of obstructive events may unmask or modify central respiratory instability rather than eliminate sleep-disordered breathing [10,11]. These interactions have important implications when considering newer therapies, which will be discussed in this review.

## 4. Current Diagnostic Tools in SRBD and New Developments

### 4.1. Current Diagnostic Tools

The gold standard for diagnosis of SRBD is polysomnography, which provides continuous monitoring throughout the sleep cycle. Key components include assessment of sleep stages using electroencephalogram (EEG) and electrooculogram (EOG), and evaluation of cardiac function via electrocardiogram (EKG) and heart rate monitoring. Respiratory status is measured through oronasal thermistors and nasal pressure sensors, while thoracic and abdominal effort belts assess ventilatory effort [12]. Oxygen saturation is monitored with pulse oximetry.

OSA is typically classified by severity based on the apnea–hypopnea score, which reflects the mean number of apneic and hypopneic episodes per hour of sleep [13]. Apnea is defined as a complete cessation of airflow for at least 10 s, while hypopnea represents a partial reduction in airflow (typically ≥30%) accompanied by either oxygen desaturation or arousal from sleep [14]. The Apnea–Hypopnea Index (AHI) serves as a standardized metric for disease burden, incorporating both the frequency and physiologic impact of disordered breathing events during sleep [15]. Severity is graded as follows: mild with AHI 5–15 events/hour, moderate with AHI 16–30 events/hour, and severe with AHI greater than 30 events/hour. Primarily, this index is obtained using polysomnography sleep testing, which remains the gold standard for both treatment and classification of severity [13].

### 4.2. Innovations in Diagnostic Tools

The current gold standard of diagnosis includes polysomnography; however, access may be limited due to high expense and time consumption, which can contribute to underdiagnosis [16]. A growing field focuses on at-home testing devices, which can reduce transportation issues, increase the level of comfort, and assess patients in their normal setting.

Peripheral arterial tonometry (PAT) provides an alternative home diagnostic testing using a device worn on the wrist and finger during sleep. PAT aims to measure changes in peripheral arterial volumes through a plethysmograph in combination with pulse oximetry and actigraphy. Arterial volume fluctuations are measured by changes in smooth muscle mediated through alpha receptors; these changes are correlated with oxygen desaturation [17]. Diagnostic accuracy is based on the severity of the patient’s OSA. Despite heterogeneity among included studies, a 2022 meta-analysis including 17 high-quality studies and 1318 participants compared PAT and polysomnography and found pooled sensitivity and specificity of 94.1% and 43.5% at an AHI threshold ≥ 5 events/hour, 92.2% and 72.4% at ≥15 events/hour, and 74.1% and 87.1% at ≥30 events/hour, respectively [18].

However, there has been a demonstrated significant misclassification with this device, particularly in patients with mild–moderate disease [17]. One cohort study with participants undergoing simultaneous PAT and polysomnography found an overall diagnostic accuracy of 53% when looking at all 3 categories of OSA, with a tendency for PAT to overestimate disease severity [17]. Overall, this study provides evidence supporting the reasonable diagnostic performance of PAT in patients who are classified as moderate-to-severe OSA. This was re-demonstrated by the 2022 meta-analysis that showed clinically significant discordance between PAT and polysomnography, particularly in mild–moderate OSA [18]. Given the substantial variability in diagnostic performance across severity thresholds, guidelines recommend confirmatory polysomnography for any patient with an abnormal PAT result. Collectively, the current evidence supports the use of PAT in patients who have a high pretest probability, and to show caution when interpreting results in patients with mild disease or when treatment depends on precise disease classification.

Several portable home monitoring systems are currently under development, allowing for monitoring within the patient’s home [19]. Proposed systems include a two-patch kit that is applied to the forehead and chin with soft, pliable material that conforms to the facial structures. In real time, a patch over the forehead can record EEG and EOG, while the chin patch records electromyography (EMG) [19]. One of the more advanced features includes convolutional neural networks, which, in real time, can quantify the sleep staging as well as detect episodes of apnea. The synthesized information can be transmitted over Bluetooth to other devices. Although this is not yet on the market, the suggested system is going through trials to improve the adhesive layer and undergo large-scale testing to confirm the accuracy of results versus the gold standard polysomnography.

Emerging technologies for diagnosing SRBD aim to improve accessibility for patients by allowing them to perform the diagnostic test in their own home or indirectly assessing metrics that may be a consequence of SRBD. For the aforementioned tests, there are multiple confounding variables that may affect the accuracy of the tests. Table 1 describes differences in diagnostic modalities available.

### 4.3. Optical Coherence Tomography

Optical Coherence Tomography (OCT) can be used as an adjunct biomarker tool to assess the extent of peripheral damage from longstanding OSA. This method uses near-infrared light to create high-resolution, cross-sectional images of retinal and choroidal microstructure. OCT can detect long-term damage due to hypoxia from OSA, specifically choroidal microvascular remodeling leading to thinning of the retinal nerve fiber layer [20]. The degree of disease provides a measure of the cumulative hypoxic damage that has occurred over time [20]. This method offers a less intrusive and rapid assessment; however, other factors such as age, history of glaucoma, and history of diabetes can confound these measurements.

### 4.4. Emerging Metrics for OSA Severity and Disease Burden

While AHI remains the most widely used metric for diagnosing OSA, it has important limitations. As a frequency-based measure, AHI quantifies the number of respiratory events/hour but does not account for the depth of oxygen desaturation, duration, or physiological consequences of each event [21]. AHI can vary substantially between nights due to factors such as body position, sleep stage, alcohol, medication, and fluid balance, leading to significant variation. These limitations prompt interest in alternative metrics that may better reflect disease burden and associated health risks.

#### 4.4.1. Physiologic Burden Metrics

One such measure is hypoxic burden (HB), which quantifies the total area under the respiratory event-related oxygen desaturation curve normalized to total sleep time [22]. Unlike AHI, HB captures both the severity and durations of oxygen desaturation, providing a more comprehensive assessment of physiologic impact. The prognostic significance of HB was shown in two prospective cohort studies for patients with OSA: the Outcomes of Sleep Disorders in Older Men (MrOS) study, which included 2743 men (mean age 76.3 ± 5.5 years), and the Sleep Heart Health Study (SHHS), which included 5111 middle-aged and older adults (52.8% women; mean age 63.7 ± 10.9 years). Across both cohorts, individuals in the highest HB quintile had significantly greater cardiovascular disease (CVD)-related mortality, with hazard ratios of 2.73 in MrOS and 1.96 in SHHS [22]. The replication of findings across two independent populations strengthens the validity and generalizability of these results. While AHI remains the cornerstone for OSA diagnosis and severity classification, these findings suggest that HB may capture aspects of disease burden that are not reflected by event frequency alone. As evidence continues to accumulate, HB may prove to be a useful adjunct for cardiovascular risk stratification and could help refine the assessment of OSA beyond traditional event-based metrics.

These findings were further supported by a subsequent analysis of the Multi-Ethnic Study of Atherosclerosis (MESA) and MrOS cohorts, which examined the relationship between OSA-related physiological burdens and incident CVD [23]. The study evaluated multiple dimensions of disease burden, including hypoxic burden, ventilatory burden, and arousal burden, in large community-based cohorts with longitudinal follow-up. Both hypoxic burden and ventilatory burden (the area under the ventilation signal during respiratory events) were independently associated with incident CVD. The adjusted hazard ratios per one standard deviation increase of 1.45 in MESA and 1.13 in MrOS [23]. In contrast, arousal burden was not significantly associated with incident CVD, highlighting the potential importance of cumulative hypoxic and ventilatory stress, rather than sleep fragmentation alone in OSA. While additional studies are needed to determine how these metrics should be incorporated into routine clinical practice, these findings further support the concept that physiologic burden may provide important complementary information beyond traditional event-based measures, such as AHI.

A related physiologic metric commonly evaluated is the arousal index, defined as the number of EEG arousals per hour of sleep. While arousal burden measures the cumulative duration of time aroused during the sleep cycle, the arousal index focuses on the total number of instances. Traditionally, the arousal index has been considered a marker of OSA severity and sleep fragmentation. However, emerging evidence suggests that its relationship with adverse clinical outcomes is more nuanced. Recent studies have demonstrated that arousal burden is not consistently associated with incident CVD outcomes, in contrast to HB [24]. The consensus from an American Thoracic Society workshop highlighted that inclusion of arousal-based hypopneas weakens the association between OSA severity and CVD risk, such that overall assessment of OSA by use of respiratory events and EEG arousals may carry less prognostic significance compared to respiratory events and substantial oxygen desaturation [24]. In fact, lower arousal indices have paradoxically been linked to an increased risk of cerebral white matter disease and atrial fibrillation, raising the possibility that diminished arousability may reflect a physiological adaptation to chronic OSA. Thus, a lower arousal index may not necessarily suggest less severe disease and can even confer poorer outcomes such as atrial fibrillation and stroke. Collectively, these findings suggest that sleep fragmentation alone may inadequately capture the cardiovascular consequences of OSA and further support the importance of considering physiologic burden alongside traditional event-based metrics.

These observations have prompted interest in metrics that better reflect the physiologic burden of OSA. One such metric is time spent below 90% oxygen saturation (T90), defined as the percentage or total minutes of sleep with oxyhemoglobin saturation below 90%. By measuring total hypoxemic time, T90 may better reflect the downstream consequences of OSA. A JACC State-of-the-Art review highlighted data from over 10,000 Canadian patients followed for a median of 68 months, demonstrating that AHI was not independently associated with mortality, whereas T90 of ≥9 min was associated with a 58% increase in all-cause mortality [25]. Thus, there is promising utility in incorporating T90 as a complementary measure of disease severity and prognosis, though the precise clinical role remains to be established. The large cohort size and extended follow-up period strengthen the clinical relevance of these observations, although the predominantly observational nature of the available evidence limits conclusions regarding causality. Nevertheless, these studies have contributed to the growing interest in oxygenation-based metrics that may better capture the physiologic burden of OSA than event frequency alone.

#### 4.4.2. Sleep Architecture and Microstructure

Beyond arousal, OSA significantly disrupts overall sleep architecture. Increasing disease severity is associated with reductions in restorative N3 (slow-wave) and REM sleep, accompanied by increased time spent in lighter N1 sleep [26]. While these changes have been long recognized, recent advances in sleep analysis may suggest that conventional sleep staging may underestimate the extent of sleep disruption. In an Artificial Intelligence (AI)-led deep learning-based study of 446 patients, conventional 30 s increments for sleep staging were shown to underestimate the degree of sleep fragmentation experienced by patients with OSA. Rather than using the 30 s increments, AI was programmed to be able to track data based on one-second intervals. By analyzing sleep on a second-by-second basis, investigators identified substantially more frequent transitions between sleep stages, revealing a stronger relationship between severe OSA and disrupted sleep compared to those detected using traditional scoring methods [27]. Compared to non-OSA controls, the association between severe OSA and fragmented sleep increased the hazard ratio of 2.90 using conventional scoring to 8.11 using the higher resolution approach [27]. Though promising, the significance of these findings is unclear in relation to changes in clinical practice. Additionally, further validation of AI models is needed before these techniques can be incorporated into routine clinical practice.

Measures of sleep microstructure may provide even greater insight into the functional consequences of OSA. For example, in young and middle-aged adults with OSA, the cyclic alternating pattern (CAP) rate and phase A3 index have demonstrated stronger correlations with cognitive impairment, as measured by the Montreal Cognitive Assessment (MoCA), and excessive daytime sleepiness, as measured by the Epworth Sleepiness Scale (ESS), than conventional measures of sleep architecture [28]. Together, these studies support the growing idea that the consequences of OSA grow beyond respiratory events and can be characterized by assessment of sleep stability and architecture.

### 4.5. Endotypes and Precision Medicine—PALM Framework

Recent advances in sleep medicine have shifted the understanding of OSA from a purely anatomic disorder toward a heterogeneous condition characterized by distinct physiologic endotypes and clinical phenotypes [29,30]. While upper airway collapsibility remains an important contributor to disease, multiple non-anatomic factors may influence disease severity and treatment response. These key endotypic traits are included in the PALM framework, which includes P—Pcrit, or upper airway collapsibility; A—arousal threshold; L—loop gain; and M—muscle responsiveness. This scale is designed to estimate the underlying mechanisms contributing to OSA severity and predict treatment response. Importantly, incorporation of endotypic traits into clinical decision-making allows treatment selection to move beyond a one-size-fits-all approach based solely on the AHI. By identifying the dominant physiologic contributor to disease, clinicians can better determine whether patients are more likely to benefit from therapies targeting airway structure, upper airway neuromuscular function, ventilatory control, or weight-related mechanisms.

The first aspect of the PALM framework focuses on Pcrit, which is a method of quantifying the critical closing pressure and represents upper airway collapsibility, or the pharyngeal pressure at which the upper airway closes during sleep. A more positive Pcrit indicates a highly collapsible airway, reflecting reduced structural stability and increased susceptibility to obstruction. Patients with elevated Pcrit often have greater benefit from interventions that directly improve airway anatomy or dilator muscle function, including maxillomandibular advancement, hypoglossal nerve stimulation, or targeted upper airway surgery [11,29]. Conversely, patients with relatively preserved airway anatomy but persistent symptoms may have less benefit from purely anatomic interventions and require evaluation for non-anatomic contributors such as ventilatory instability or impaired muscle compensation.

The second aspect of the PALM framework, arousal threshold, refers to the level of respiratory effort or physiologic disturbance required to trigger awakening from sleep. Individuals with a low arousal threshold awaken in response to relatively minor respiratory disturbances, resulting in sleep fragmentation and reduced opportunity for airway dilator muscle activation. Alternatively, patients with a higher arousal threshold may tolerate greater levels of airway obstruction before awakening but may experience more severe oxygen desaturation. Identification of arousal threshold may help guide therapy selection, as certain patients with low thresholds may respond to interventions that stabilize sleep continuity or modify respiratory drive [11,29]. For these patients, therapies aimed at reducing sleep fragmentation or increasing airway stability without provoking excessive respiratory effort may be particularly valuable. In contrast, patients with high arousal thresholds may require interventions that address the underlying obstruction because prolonged obstructive events may occur before arousal-mediated recovery mechanisms are activated.

Ventilatory control instability, measured in loop gain, describes the responsiveness of the respiratory control system to disturbances in ventilation and is the third phenotype in the PALM framework. High loop gain reflects an exaggerated ventilatory response to changes in carbon dioxide and oxygen levels, producing fluctuations in breathing that may perpetuate both central and obstructive respiratory events. Patients with elevated loop gain may demonstrate persistent disease despite correction of upper airway anatomy and may benefit from therapies targeting ventilatory control, including adaptive positive airway pressure strategies or other approaches intended to stabilize respiratory drive [11,31]. Recognition of elevated loop gain may therefore explain treatment failure after anatomically directed therapies and identify patients in whom therapies that stabilize ventilatory control may provide greater benefit. This is particularly relevant in patients with treatment-resistant CSA or mixed obstructive and central respiratory conditions.

The last characteristic in the PALM framework focuses on upper airway muscle responsiveness. It describes the ability of pharyngeal dilator muscles, particularly the genioglossus, to activate during sleep and oppose airway collapse. Individuals with impaired muscle compensation may experience obstruction despite relatively normal airway anatomy, whereas those with preserved muscle responsiveness may maintain airway patency despite increased airway collapsibility. This physiologic trait provides a rationale for therapies such as hypoglossal nerve stimulation, which enhances genioglossus activation during sleep [11]. Assessment of muscle responsiveness may help identify patients whose obstruction is primarily related to impaired neuromuscular compensation rather than fixed structural narrowing, allowing selection of therapies designed to augment airway dilator activity rather than solely modify anatomy.

### 4.6. Patient-Centered Outcome Measures

Importantly, objective physiologic measures do not always correlate directly with a patient’s perceived disease burden. Several other validated patient-reported outcome measures (PROMs) have been developed to capture dimensions of OSA burden that are not reflected by the AHI. Commonly used PROMs assess complementary aspects of health status and treatment response.

The Functional Outcomes of Sleep Questionnaire (FOSQ-30) evaluates the impact of sleepiness on daily activities. Scores range from 5 to 20, with scores ≥ 17.9 considered normal. The minimal clinically important difference (MCID) is one point [32]. The sensitivity of this questionnaire makes it ideal for clinical trials for comparison of different treatment modalities. A shortened version of this questionnaire with only 10 items (FOSQ-10) has been seen to perform very similarly to the full version. Given this abbreviated length, it would be much better used for clinical monitoring in an outpatient setting [33]. The Sleep Apnea Quality of Life Index (SAQLI), the only OSA-specific quality-of-life instrument developed with input from both patients and physicians, evaluates daily functioning, social interactions, emotional functioning, and symptoms [34]. Since this modality addresses treatment-related symptoms, it is best suited for comparing interventions with different side effect profiles. More general measures include the EQ-5D-5L, which measures general health-related quality of life, and the Glasgow Benefit Inventory, which quantifies changes in quality of life following surgical intervention on a scale from −100 to +100 [32]. The EQ-5D-5L is often more appropriate for cost-effective analysis since it can generate utility scores. These scores can then, in turn, be used to calculate quality-adjusted life years (QALYs). However, there is a significant ceiling effect in sleep disorder populations, or patients stating that they have the highest possible score with continued sleep impairment [35]. There is no true PROM that is preferred, and the choice of PROM is dependent on the clinical or research context. Together, these instruments provide a more comprehensive assessment of treatment effectiveness by capturing outcomes that are meaningful to patients but may not be reflected in traditional respiratory event indices.

Multiple studies have shown the significant effect of using PROMs to determine treatment. In a systematic review performed by the U.S. Preventive Services Task Force, positive airway pressure (PAP) therapy produced statistically significant but clinically modest improvements in quality of life. Specifically, treatment improved the physical component score of the Short Form-36 (SF-36), a widely used measure of physical health status and daily functioning, by an average of 1.53 points. This remained below the established MCID of 4–7, suggesting that the average improvements in sleep-related quality of life were modest overall [36]. Notably, these benefits were more pronounced among patients with greater baseline symptom burden, particularly those with an ESS score of 10 or higher.

Collectively, these findings highlight the importance of incorporating PROMs into OSA assessment, as they provide a more comprehensive evaluation of treatment effectiveness and patient well-being than respiratory event indices alone.

## 5. Traditional Approaches to Treatment

### 5.1. Conservative Management

Conservative management of SRBD aims to control symptoms, improve sleep quality, and reduce long-term complications. Core strategies focus on reducing upper airway obstruction and improving sleep quality, often alongside definitive therapy, such as positive airway pressure (PAP), particularly in those with OSA.

Patients with OSA and modifiable risk factors should receive individualized behavioral recommendations. Obesity remains one of the most prominent risk factors, making weight reduction a central therapeutic target. A 2024 systematic review and meta-analysis incorporating 27 studies and 1400 participants illustrated that a 20% reduction in body mass index (BMI) was associated with a 57% reduction in the severity of OSA, as measured by the AHI [37]. The study evaluated the relationship between weight reduction and OSA severity and utilized pooled statistical analysis across multiple studies, strengthening the applicability of the findings to current clinical practice. A longitudinal study found that a 10% reduction in body weight correlated with a 26% decrease in AHI, underscoring the clinical impact of weight loss [38]. The consistent relationship observed between weight reduction and improvement in AHI across the included studies further supports the clinical applicability.

This improvement may be achieved by lifestyle modifications, such as increasing physical activity or diet modification. In patients with type 2 diabetes, the Sleep AHEAD study reported that the 10-year remission rate of OSA was 34.4% with lifestyle modifications, whereas those receiving education and diabetic management had a remission rate of 22.2% [39]. This evidence supporting lifestyle modification is moderate-to-strong in quality, as the model was a prospective randomized controlled trial with clearly defined intervention and control groups with a long follow-up interval of 10 years. With clear endpoints and persistent benefit over prolonged periods, these findings reinforce the role of structured lifestyle intervention as a pillar of treatment.

Optimizing sleep habits is another core tenet of SRBD management. The severity of AHI has been associated with sleep position and is a simple and easily modifiable factor in addressing SRBD management. Patients should aim to maintain a non-supine sleep position, as the AHI can be nearly twice as high in individuals with normal BMI who sleep on their back compared to their side [40]. The postural effect became less pronounced with increasing BMI. Patients should also be advised to avoid or reduce alcohol consumption before sleep, as alcohol can exacerbate upper airway collapse and worsen sleep-disordered breathing. A meta-analysis showed that in patients who consume alcohol, the prevalence of OSA was 25% higher, and they had longer duration of apnea with lower oxygen saturations [41].

PAP is the foundation of conservative management of SRBD. The American Academy of Sleep Medicine (AASM) recommends PAP therapy for all patients with OSA, given its efficacy in reducing AHI, improving daytime sleepiness, and enhancing quality of life [42]. PAP aims to prevent upper airway collapse during sleep by maintaining an intraluminal pressure exceeding that of the surrounding pressure, thereby preventing collapse of the upper airway during sleep. Several modalities of PAP exist, including continuous PAP (CPAP), bilevel PAP (BiPAP), and autotitrating PAP (APAP). CPAP remains first-line therapy in SRBD; BIPAP may be preferred in patients with concurrent hypoventilation syndromes [37].

### 5.2. Pharmacological Interventions

Current clinical guidelines do not consistently identify effective pharmacologic therapies for SRBD, leaving a large gap in adequate patient care if they are unable to tolerate CPAP. However, there are studies exploring medications for off-label use in reducing the disease severity by targeting specific pathways involved in upper airway control.

Some studies involving norepinephrine reuptake inhibitors, including atomoxetine or reboxetine, with an antimuscarinic medication such as oxybutynin, have been shown to reduce the AHI. When patients are awake, the excitatory effects of norepinephrine help maintain upper airway muscle tone. During sleep, however, norepinephrine levels naturally decrease, leading to relaxation of pharyngeal dilator muscles. Conversely, increased muscarinic activity during sleep further reduces the activation of upper airway muscles. Together, the effects of these pathways lead to decreased muscle tone during and can contribute to SRBD by causing partial airway obstruction. Thus, targeting these pathways with pharmacologics offers a potential treatment for those who cannot tolerate CPAP.

A phase II randomized controlled trial compared AD036 (fixed-dose combination of atomoxetine 80 mg and oxybutynin 5 mg) with atomoxetine 80 mg or placebo [43]. The primary endpoint involved time spent hypoxic with an SpO2 < 90%. Among patients with moderate pharyngeal collapsibility, AD036 had significant improvement in OSA severity with a reduction in AHI by greater than 50% relative to placebo or untreated controls [43]. Evidence remains limited by the selective patient population, namely those with moderate pharyngeal collapsibility, which may limit the applicability to the broader and increasingly heterogeneous population of patients with OSA. There was no analysis provided for long-term efficacy or safety, which would be recommended before moving forward with integration into treatment regimens.

One proposed mechanism for the development of OSA relates to decreased serotonergic activation of upper airway motor neurons, leading to collapse of the upper airway. Subsequently, studies have evaluated medications targeting serotonergic pathways. A prospective trial evaluated fluoxetine, which delivers central serotonergic stimulation, and ondansetron, which activates peripheral serotonin receptor antagonists [44]. This combination resulted in a mean reduction in AHI by 40%, without notable improvement in daytime sleepiness [44]. The evidence supporting this approach is limited due to its small sample size of 25 adults with a short treatment duration of 28 days without long-term follow-up data. The absence of subsequent validation studies limits confidence in the durability of these findings. Therefore, this therapy should be considered investigational, and larger randomized controlled trials are needed before its routine use can be recommended.

Medications such as fluoxetine alone and protriptyline have been investigated, which are expected to decrease the duration of time spent in the rapid-eye movement (REM) cycle of sleep. These studies found a decrease in the number of apneic and hypopneic events in non-REM sleep. However, there was no consistent response and no decrease in desaturation or arousals, leaving these medications without significant improvement in sleep architecture [45]. Mirtazapine has been shown in animal models to decrease central apnea during non-REM and REM cycles, but it has not been replicated in human models and has led to weight gain, which may have contributed to worsening disease [46].

Several new ongoing clinical trials are advancing the pharmacological approach to SRBD. The AD109 (aroxybutynin and atomoxetine combination pill) has gone into a phase III trial after showing significant reductions in AHI in the phase 2 MARIPOSA trial [47]. The phase II randomized controlled trial showed a placebo-adjusted reduction in AHI by 42% and overall OSA severity. Secondary measures showed improvements as well, including oxygen desaturation indices, nocturnal hypoxic burden, and patient-reported measures. The consistent improvements across multiple physiologic measures support the advancement to phase III evaluation.

Tirzepatide is the first drug approved by the FDA for moderate-to-severe OSA in adults following the SURMOUNT-OSA trial in which the treatment was shown to reduce AHI by 58.7%, corresponding to a decrease in AHI by 29.3 events/hour over 52 weeks [48]. Notably, there was significant improvement in patients’ self-reported sleep function, sleep disturbance, body weight, inflammatory markers, and hypoxic burden regardless of PAP usage. As a large multicenter randomized controlled trial, SURMOUNT-OSA provided strong evidence supporting metabolic therapy as a disease-modifying approach to OSA management rather than solely symptomatic treatment.

The success of tirzepatide has accelerated interest in additional metabolic therapies such as Eloralintide, a selective amylin receptor agonist. This drug is currently in a phase 3 study, called ENLIGHTEN-3, measuring its relationship with OSA and overweight patients. Building on this, other metabolic agents are also under investigation, such as the SGLT-2 inhibitor Bexagliflozin, currently in phase IV trials [49]. Other new medications not mentioned previously, which are currently under investigation, include acetazolamide, trazodone, and dronabinol.

### 5.3. Mandibular Advancement Devices

Oral devices such as mandibular advancement devices (MADs) provide a viable alternative for patients unable to tolerate CPAP. These devices primarily act by advancing the mandible anteriorly, stabilizing and reducing lateral collapse in the velopharyngeal area [50]. MADs can be a first-line option for patients with mild–moderate OSA and are considered in patients with severe OSA who decline CPAP [23]. Before device creation, patients should undergo a dental evaluation to assess mandibular-maxillary relationships and the range of motion of the mandible to create a custom device [51].

Studies have proven that oral appliances have been comparable in improving cardiovascular mortality, quality of life, and neurocognitive function when compared to traditional PAP therapy. In a randomized controlled crossover trial directly comparing CPAP with MADs, both therapies produced similar improvements in blood pressure, daytime sleepiness, driving performance, and quality of life despite CPAP producing greater reductions in AHI [52]. The crossover randomized design and direct comparison of established therapies strengthened the clinical applicability of these findings. When comparing the effectiveness of the treatments, a 2016 meta-analysis with data from 51 randomized controlled trials with over 3700 participants found that CPAP led to a decrease in AHI by 25.4 events/hour, while MADs were found to have a reduction of 9.3 events/hour [53]. Collectively, these studies suggest that although CPAP remains more effective in reducing AHI, MAD therapy may still provide symptomatic and functional improvement for patients.

Preliminary studies focused on combination therapy with CPAP have found that oral appliances used in combination with PAP therapy lower the resistance of the upper airway, in turn lowering the pressure required to keep the airway patent [54]. Although it has not yet been adopted into guidelines and will require a larger-scale evaluation in order to be a widespread practice, this provides a useful approach to CPAP treatment. While CPAP remains superior in reducing AHI, MADs have been found to have a higher adherence rate. MADs, on average, had 6.5 h of compliance nightly compared to CPAP, which was an average of 5.2 h nightly [55]. This makes MAD a viable option for patients with mild-to-moderate disease who are intolerant of CPAP therapy to offer some improvement in AHI and symptomatic control. An adverse effect of MADs can be the development of temporomandibular joint (TMJ) pain; therefore, patients should be evaluated for TMJ pain before initiation of therapy and educated about the risk of developing TMJ-related symptoms.

## 6. Overview of Surgical Interventions for SRBD

### 6.1. Indications for Surgery

In addition to lifestyle modifications and PAP therapies, surgical intervention represents an important treatment option for selected patients with SRBD. Over time, numerous surgical approaches have been developed and refined, most of which target upper airway obstruction to prevent airway collapse. As a result, surgical management is primarily applicable to OSA, which is fundamentally an anatomic disorder. While certain surgeries may provide indirect benefit for CSA or sleep-related hypoventilation, they mainly focus on modifying underlying contributing conditions rather than correcting the fundamental physiologic defect. An overview of surgical modalities is highlighted in Table 2.

Appropriate surgical selection includes a comprehensive evaluation of disease severity, the location of airway collapse, patient anatomy, and relevant comorbidities. While surgery plays a central role in the management of OSA due to its anatomic basis, other forms of SRBDs are typically managed with ventilatory support, neuromodulation, or targeted treatment of the associated systemic disease. Overall, surgical therapies are generally reserved for patients who fail conservative treatments such as PAP therapy and mandibular advancement devices.

Guidance from the 2021 AASM Clinical Practice Guideline provides recommendations regarding surgical referral for patients with OSA [56]. The guideline supports referral for patients with a BMI of greater than 40 kg/m^2^ who are unwilling or unable to tolerate PAP due to pressure side effects. It favors referral of patients with a BMI of over 35 kg/m^2^ for a sleep surgeon as well as a bariatric surgeon to optimize modifiable risk factors [56]. Additionally, anatomic features that suggest a high likelihood of benefit from surgical referral, such as tonsillar hypertrophy and maxillomandibular abnormalities, should prompt consideration of surgical evaluation.

Increasingly, surgical candidacy is determined using a precision medicine framework rather than AHI alone. Although disease severity remains important, surgical decision-making now also incorporates anatomic phenotype, patterns of airway collapse identified on DISE, and physiologic endotypes described by the PALM framework. Patients with a highly collapsible upper airway (elevated Pcrit) and favorable anatomic targets may derive substantial benefit from airway reconstruction, whereas those with prominent ventilatory instability (high loop gain) or other non-anatomic contributors may experience persistent disease despite anatomically successful surgery. Accordingly, surgical evaluation emphasizes identifying the dominant mechanism of obstruction and matching interventions to patient-specific anatomy and physiology rather than relying solely on event frequency measured by AHI.

### 6.2. Role of DISE in Surgical Planning

Another major innovation in determining the need for operative intervention is drug-induced sleep endoscopy (DISE). This procedure can identify collapse patterns that are not seen while awake. This includes visualization of the upper airway, specifically anteroposterior and pharyngeal wall collapse, and determining if it is a complete or partial obstruction [57]. Evaluation is performed through the VOTE classification system. Scoring is graded by level of obstruction at each site as 0 (no obstruction—no vibration), 1 (partial obstruction—vibration), 2 (complete obstruction—collapse), or X (not visualized) [58]. DISE also evaluates specific patterns of collapse at each level, which are predictive of surgical outcomes. Complete circumferential collapse at the velum and complete anterior–posterior collapse at the tongue base are associated with poor surgical response, while grade 3–4 tonsillar hypertrophy and anterior–posterior partial collapse at the velum predict better outcomes following surgical intervention [59].

Despite its growing role in surgical planning, DISE remains limited by the subjective nature of interpretation. Studies evaluating interobserver reliability have demonstrated only fair-to-moderate agreement across commonly used scoring systems, with variability observed not only in grading severity but also in identifying the primary site pattern of airway collapse [60]. More recently, Mitsikas et al. compared several commonly used DISE classification systems and found that reliability varied considerably depending on the scoring method employed, suggesting that some of the observed variability may stem from the lack of a universally accepted grading system rather than differences in clinician expertise alone [61]. These findings emphasize the need for greater standardization and more objective methods of DISE interpretation. Reproducibility of DISE findings across institutions remains variable, which may influence both surgical decision-making and reported treatment outcomes.

An additional challenge relates to the effect of sedation on the upper airway dynamics. Because DISE attempts to reproduce physiologic sleep, both the sedative agent and the depth of sedation may influence the observed pattern of obstruction. When comparing DISE with natural sleep and drug-induced sleep with midazolam, there was a significant difference in the location and degree of upper airway collapse [58]. Furthermore, different sedative agents produce distinct neurophysiologic states and may influence upper airway muscle tone to varying degrees. For example, dexmedetomidine is often favored because it will predominantly induce an NREM-like state and preserve respiratory drive compared to other agents. However, OSA severity and patterns of airway collapse can vary substantially across sleep stages, particularly during REM sleep, raising the possibility that some obstruction patterns may be under- or overrepresented depending on the sedation protocol utilized [62]. Recognizing these limitations, expert consensus statements have emphasized standardized sedation protocols and cautious interpretation of DISE findings within the broader clinical context. Despite expert consensus recommendations, considerable variability persists regarding sedative agent selection, dosing, and depth of sedation, and no universally accepted sedation protocol currently exists. Until a universally accepted protocol is established, clinicians should interpret DISE findings as a representation of upper airway behavior, rather than a definitive characterization of airway obstruction.

AI-assisted DISE analysis may help address several of these limitations. Deep learning models trained on DISE video frames scored a mean F1 score of 70% across all VOTE sites (V: 85%, O: 72%, T: 57%, E: 65%) with a particularly strong performance in the assessment of velum collapse [63]. Importantly, model performance remained consistent across clinicians and institutions, suggesting improved reproducibility compared with conventional subjective interpretation. Similarly, Chang et al. developed an AI-assisted system that accounted for depth-related imaging variation and demonstrated improvements in grading accuracy within anatomically challenging regions from approximately 50% to 78% [64]. The potentially greatest advantage of AI-assisted DISE analysis is that it not only improves accuracy but also its ability to provide consistent assessments across clinicians and institutions reduces interobserver variability and facilitates a more standardized approach to DISE interpretation. While these technologies remain investigational, they offer a promising avenue toward more objective and standardized DISE assessment, with the potential to improve both patient selection and surgical planning.

Beyond identifying the location of airway collapse, DISE has become a cornerstone of phenotype-driven surgical planning. Contemporary algorithms use DISE findings to determine whether obstruction is predominantly retropalatal, lateral pharyngeal wall, tongue-base, or multilevel in origin. This information helps guide the selection of specific surgical procedures. The goal is to enable an individualized plan for surgical intervention rather than a one-size-fits-all method. While the evidence supporting a consistent outcome of DISE is mixed, the ability to identify individual architecture, reduce unnecessary procedures, and guide targeted intervention has made it an essential component of OSA management.

### 6.3. Role of Surgery in PAP-Intolerant Patients

It is important to contextualize surgical outcomes against the well-documented challenges in CPAP adherence. Estimates suggest that 46 to 83% of patients demonstrate nonadherence to CPAP therapy, and up to 50% of patients experience side effects from their CPAP device [65]. Although surgery does not uniformly eliminate OSA, it may offer durable modifications to anatomy that reduce disease burden in patients unable to tolerate device-based therapy.

These recommendations are particularly relevant given real-world adherence challenges with PAP therapy. Large database analyses report short-term adherence of PAP therapy at 75%, with other sources estimating adherence as low as 54% [66]. These findings underscore the substantial number of patients for whom PAP therapy may be suboptimal or unsustainable. For this subset of patients, surgical intervention may represent a viable adjunct or alternative therapy for a significant subset of patients with OSA. Although patients who undergo surgery may still require PAP therapy postoperatively, surgical modification can reduce the pressure requirements needed to maintain airway patency. This, in turn, can decrease perceived adverse effects and improve patient comfort. Surgical evaluation should be integrated into a multi-modal approach to SRBD management alongside PAP therapy.

### 6.4. Traditional Surgical Approaches

Surgical interventions for obstructive sleep apnea can be broadly classified into resective, non-resective, and skeletal (facial reconstruction) techniques. Resective procedures involve removing excess or obstructive tissue to enlarge the airway and increase airflow. Nonresective procedures focus on repositioning or stiffening airway structures to reduce collapsibility. Skeletal structures alter craniofacial anatomy to increase upper airway dimensions and address multilevel obstruction. Each category targets distinct anatomic mechanisms of airway compromise and varies substantially in invasiveness, efficacy, and durability of outcomes.

Modern surgical management increasingly employs multilevel treatment algorithms that recognize OSA as a heterogeneous disorder involving different patterns of airway collapse. Rather than selecting a single operation for all patients, surgeons often combine procedures that target multiple levels of obstruction identified during preoperative evaluation. Patient-specific planning incorporates craniofacial anatomy, body habitus, DISE findings, symptom burden, and expected treatment goals. This evolution from procedure-based to phenotype-based decision-making reflects a broader movement toward precision medicine in sleep surgery.

Surgical success in obstructive sleep apnea is most commonly assessed using changes in the AHI. Historically, surgical “success” has been defined as a reduction in AHI of at least 50% from baseline, often with a postoperative AHI < 20 events/hour. However, Elshaug and colleagues have proposed more stringent criteria to define “cure”, recommending an objective cutoff of ≤ 5 or ≤10 events/hour [67]. Although these are stricter thresholds, they may reflect true normalization of breathing during sleep and may be appropriate to separate those who will truly see resolution of sleep apnea-related sequelae. However, it should be noted that patients who do not meet the criteria for cured disease will often have decreased pressure settings of their CPAP after surgery, potentially improving patient comfort and compliance.

#### 6.4.1. Palatal and Pharyngeal Soft Tissue Procedures

Introduced in the United States in 1981, uvulopalatopharyngoplasty (UPPP) quickly became the most commonly performed surgical technique for treating soft tissue-related upper airway collapse in OSA [68,69]. UPPP enlarges the upper airway by removing and reshaping excess tissue at the back of the mouth and throat, allowing for improved airflow during sleep.

UPPP remains the most frequently performed surgical procedure for OSA; however, its success rates vary widely depending on the definition applied [70]. When strict cure criteria are applied, as described above, only 24% of patients achieve an AHI < 5, and 33% have an AHI of <10 at six-month follow-up [70]. Numerous modifications of UPPP have been trialed and implemented, but the nature of surgery makes it difficult to have comprehensive, blind comparisons.

Randomized trials evaluating UPPP with tonsillectomy demonstrate meaningful improvements in OSA severity in appropriately selected patients. In the SKUP3 randomized controlled trial, patients undergoing surgery experienced a 60% reduction in AHI compared to 11% in the control group [71]. Mean AHI improved from 53.3 preoperatively to 21.1 events/hour postoperatively, representing a significant reduction in disease burden, though disease severity would still fall under the moderate apnea severity range [71]. The study’s design and use of polysomnographic outcome measures strengthen the relevance of the data; however, generalizability is limited because the patient population was younger, most had a BMI of <30 kg/m^2^, and it excluded patients with Friedman stage III or severe comorbidity. This does highlight the need for careful surgical selection and patient-specific anatomic evaluation when considering upper airway surgery.

UPPP with tonsillectomy appears to be especially promising for those with tonsillar hypertrophy, highlighting the need to individualize the surgical approach [72]. These findings illustrate an important principle of precision sleep surgery: that favorable anatomic phenotypes are often stronger predictors of success than baseline AHI alone. Patients with prominent tonsillar hypertrophy, retropalatal obstruction, or lateral pharyngeal wall collapse generally achieve greater benefit from palatal procedures than those whose disease is driven primarily by tongue-base collapse or non-anatomic physiologic factors. Patient selection increasingly focuses on identifying the predominant site and mechanism of obstruction before selecting a specific surgical intervention. In a prospective multicenter study evaluating adults with tonsillar hypertrophy and OSA, mean AHI dropped from 33.7 to 15.4 events/hour, and 65% of participants no longer required additional treatment for OSA. The study’s focus on patient selection and consistent post-operative improvement further supports the ideology that anatomic phenotype strongly influences surgical success. This led to a subsequent update of guidelines in 2022 to include strengthened recommendations supporting combined tonsillectomy and UPP in carefully selected patients with favorable anatomy and PAP intolerance [73].

Multilevel surgical approaches have also been evaluated in randomized settings. The SAMS randomized controlled trial compared adults undergoing modified UPPP and radiofrequency tongue reduction to those receiving continued medical management [32]. At six-month follow-up, those who received palatal and tongue reduction surgery had a drop in mean AHI from 47.9 to 20.8 events/hour, as well as significant improvements in patient-reported daytime sleepiness [32]. The study also evaluated multilevel surgery targeting both palatal and tongue base obstruction, reflecting a more individualized and anatomically directed surgical strategy compared with an isolated upper airway procedure. These findings support the efficacy of multilevel airway surgery in select patients with moderate-to-severe OSA and contribute to the growing shift toward phenotype-driven surgical management. However, interpretation remains limited by the inability to blind patients, the lack of generalizability, and the persistence of residual moderate OSA despite postoperative improvement in AHI.

#### 6.4.2. Modifications to UPPP

Over time, UPPP has undergone numerous modifications to address limitations in outcomes and better target specific mechanisms of airway obstruction.

Patients with OSA are found to have a more collapsible lateral wall compared to that of a healthy person, making it a predominant anatomic contributor to airway narrowing in OSA [74]. In response, lateral expansion pharyngoplasty, first described by Calahi in 2003, was developed as a modification of UPPP [75]. This technique supports the lateral pharyngeal wall by repositioning the pharyngeal muscles, resulting in improved airway stability and greater sleep improvement. However, postoperative dysphagia limited its broader adoption.

These limitations prompted the development of expansion sphincter pharyngoplasty (ESP), which more effectively addresses lateral wall collapse during inspiration [76]. This technique functions by isolating and rotating the palatopharyngeus muscle, resulting in greater superior anterior tension on the lateral pharyngeal wall to improve airway stability.

Subsequent modifications include the barbed reposition pharyngoplasty (BRP), which was introduced as less invasive, more economical, and technically accessible [77]. This technique works via the use of barbed sutures to reposition and suspend the soft palate and lateral pharyngeal walls without the need for knots. Multiple studies have supported the safety and efficacy of BRP, though barbed suture extrusion remains a known complication [78,79]. Since 2020, studies evaluating the rate of extrusion in BRP procedures have found it to be relatively high, occurring in 18.4% of all BRP procedures [80]. Patients enrolled in the study received a pre-operative and post-operative polygraph to evaluate the effects of suture extrusion. The results showed that extrusion did not adversely affect patients’ perceived quality of life or the follow-up polygraphy outcomes, indicating that patients’ sleep patterns were not negatively affected by extrusion [80].

Laser-assisted uvulopalatoplasty (LAUP) reduces AHI to below 10 events/hour in 50% of cases [81]. However, a large proportion of cases were found to have postoperative side effects, including dysphagia, uvular necrosis, or velopharyngeal insufficiency leading to nasal regurgitation. This was introduced in the 1990s and performed in an outpatient setting, but has lost popularity due to the increased risk for post-operative complications.

#### 6.4.3. Skeletal and Multilevel Airway Surgery

Maxillomandibular advancement (MMA) is a major orthognathic surgical procedure that advances both the upper (maxilla) and lower (mandible) jaws forward, thereby increasing the dimensions of the airway while reducing the probability of airway collapse [82,83]. By advancing both the mandible and maxilla, MMA enlarges the retropalatal and retrolingual airway, and studies have demonstrated a positive correlation between the degree of advancement and airway dimension. A systematic review and meta-analysis incorporating 13 studies and 200 patients evaluating pharyngeal airway volume in those treated with MMA demonstrated that for approximately 1 mm of expansion of these regions, there is a corresponding increase in airway dimensions by 0.5 mm, and a reduction in AHI by 3.58 events/hour [84]. Although limited by a small sample size, the consistent association between airway expansion and reduction in OSA supports the proposed mechanism of action. These results support the use of objective craniofacial and airway measurements in preoperative surgical planning and patient selection for MMA.

MMA represents one of the clearest examples of phenotype-directed surgery because it is particularly effective in patients with craniofacial restriction, retrognathia, micrognathia, or severe multilevel airway narrowing. These patients are ideal candidates for structural airway expansion. In contrast, those whose residual disease is driven predominantly by ventilatory instability or other non-anatomic traits may require adjunctive therapies despite technically successful skeletal advancement.

MMA demonstrates the highest success rates among contemporary surgical interventions for OSA, excluding tracheostomy, which bypasses the upper airway completely. In comparison to isolated soft tissue procedures, MMA addresses multilevel airway obstruction and has consistently demonstrated the highest success rates among surgical treatments for OSA. A 2010 meta-analysis incorporating 22 studies and over 600 patients reported a reduction in mean AHI from 63.9 to 9.5 events/hour following surgery [85]. However, limitations do include the predominance of observational surgical series that include heterogeneity of operative technique and patient selection, which makes it difficult to apply to the broader OSA population. More recent evidence has continued to expand on these findings. A 2025 meta-analysis with 31 studies and 1597 patients reported a considerable and consistent reduction in AHI across institutions. This included a mean reduction in AHI of 41.87 events/hour, improvement in oxygen saturation nadir by 6.29%, and reduction in Epworth Sleepiness scale scores by 8.69 points [86]. This study included a substantially larger patient population with multicenter data, which improved the strength of and applicability to current surgical practice. The consistent postoperative improvement amongst multiple meta-analyses strengthens the clinical applicability of MMA as an effective surgical strategy for severe OSA.

With MMA, a reported 86% of cases achieved surgical success [87]. Cure rates are defined as AHI < 5 events/hour, reported after MMA in 43–47% of cases [88]. The optimal distance for advancement remains a topic of debate, though recent studies suggest a distance of around 6–10 mm is effective. Overall, individual adjustments for length are recommended for aesthetic and individual anatomy.

The mean hospitalization duration for this procedure is approximately 3.5 days [89]. Mouth opening amplitude is significantly decreased during the first two weeks, but gradually improves over three months, with most patients returning to regular functional status within 2–10 weeks [87]. Mild-to-moderate lateral pharyngeal wall edema occurs in most cases, peaking at 48–72 h post-operatively. Significant facial edema is common, with mean increases of 4.53–7 mm in various facial measurements, which will also gradually subside [89]. Patients will require monitoring of airway safety with these complications in mind.

Complications of this procedure include hemorrhage, infection, hardware extrusion, and jaw stiffness. The most significant adverse effect is injury of the lower facial nerve occurring in about 83% of patients, commonly due to stretching or iatrogenic injury of the inferior alveolar nerve [86]. This most often resolves in 85–90% of patients by 6–12 months post-operative [87]. One unconventional outcome showed an effect on the perception of face aesthetics. In one study, 95% of external evaluators judged postoperative changes as positive or neutral, with mean esthetic scores improving significantly [90]. Numerous patients have had facial rejuvenation through a ‘reverse facelift’ effect due to the way the facial bones are rearranged, rather than having the skin of the face pulled back [88].

Due to its invasiveness and associated recovery, MMA is typically reserved for patients with moderate-to-severe OSA, craniofacial abnormalities, or persistent disease despite prior therapies [86]. This includes patients who are unable to tolerate CPAP or remain refractory after other surgical interventions.

#### 6.4.4. Tongue Base and Hypopharyngeal Procedures

Several surgical techniques target tongue base collapse, a common contributor to hypopharyngeal obstruction. Mandibular osteotomy with genioglossus advancement repositions the genioglossus muscle anteriorly to prevent posterior tongue collapse during sleep, referred to as Genioglossus Advancement and Hyoid Myotomy (GAHM). It is often combined with hyoid suspension to further stabilize the hypopharyngeal airway [91]. Inferior sagittal mandibular myotomy and suspension represents a more comprehensive modification of this approach, involving an inferior sagittal split of the mandible with anterior repositioning and suspension of the genioglossus-hyoid complex to achieve more sustained enlargement of the hypopharyngeal airway. By addressing both tongue base position and hyoid stability, this technique aims to reduce dynamic airway collapse during inspiration. While these procedures can improve airflow and reduce AHI, outcomes are generally modest compared with MMA.

Several procedures, including nasal reconstruction, hyoid suspension, and radiofrequency ablation of upper airway structures, are generally non-curative when performed alone [81]. These adjunctive procedures may improve airway resistance or contribute to multilevel strategies, but rarely normalize AHI alone.

#### 6.4.5. Definitive Surgical Intervention

Tracheostomy remains the most definitive surgical treatment for OSA, bypassing upper airway obstruction entirely [92]. However, creating a surgical opening in the neck has a profound impact on the quality of life and long-term care requirements. For this reason, it is reserved for patients with refractory and/or life-threatening obstructive sleep apnea. Tracheostomies also have utility for sleep-related hypoventilation disorders, particularly in patients with neuromuscular disease requiring lifelong ventilatory support [92].

#### 6.4.6. Adjunctive Surgical Interventions

Bariatric surgery represents an indirect surgical option for SRBD, especially in patients with obesity hypoventilation syndrome and obesity-related OSA. Procedures such as sleeve gastrectomy and Roux-en-Y gastric bypass can result in significant weight loss, which is associated with improvements in OSA severity [93]. Gas exchange and oxygen exchange do improve post-operatively, though effects are not clearly seen until the patient achieves a weight reduction of more than 20% [93].

Nasal procedures, including septoplasty, turbinate reduction, and polypectomy, are not curative for OSA when performed in isolation. However, they may reduce nasal resistance, improve subjective sleep quality, and enhance tolerance to PAP therapy. Tonsillectomy and adenoidectomy remain first-line for pediatric OSA and may also benefit select adults with hypertrophic tonsils.

### 6.5. Minimally Invasive Surgeries

#### 6.5.1. Pillar Implant

Pillar implants are one of the first major minimally invasive interventions used in OSA. These devices consist of polyethylene terephthalate rods inserted into the soft palate to reduce vibration and airway collapsibility [94]. The procedure involves the placement of three rods along the midline soft palate with local anesthesia.

A meta-analysis incorporating 7 studies and 714 patients demonstrated moderate efficacy in treating both snoring and mild-to-moderate OSA. Specifically, pillar implants were associated with a significant reduction in snoring intensity (standardized mean difference −0.591) [95]. However, the evidence base remains relatively limited compared with other OSA interventions, as the majority of the included studies involved patients with mild-to-moderate disease and short-to-intermediate follow-up periods. Consequently, pillar implants are primarily considered for carefully selected patients with bothersome snoring or mild OSA rather than those with more severe disease requiring substantial reductions in AHI.

Initial treatment response appears to be the strongest predictor of long-term outcomes. Patients who demonstrated improvement at 90 days maintained these gains at extended follow-up, whereas non-responders showed no significant change in AHI or daytime sleepiness over time [96]. However, even among initial non-responders, reductions in snoring persisted at longer-term follow-up, suggesting greater durability of subjective symptom improvement compared with objective polysomnographic measures. These findings have helped define the role of pillar implants in contemporary practice, limiting their use primarily to carefully selected patients with primary snoring or mild OSA rather than individuals with more severe disease.

The major complication from this procedure is extrusion, or the protrusion of the implants from the soft palate. Pooled extrusion rate across studies was 9.3% (95% CI, 7.0–12.2%), indicating a modest but relevant clinical risk with the procedure [95].

#### 6.5.2. Radiofrequency Ablation (RFA)

Radiofrequency ablation (RFA) works by using a probe to deliver energy, typically 600–700 joules per lesion, to create submucosal fibrotic tissue. This fibrotic tissue stiffens redundant upper airway soft tissue while sparing the overlying mucosa and adjacent structures [94]. RFA became a popular technique due to reduced recovery time, pain, and morbidity compared to normal resection. More recently, Emara et al. evaluated multilevel RFA of the tongue base, soft palate, and inferior turbinates in patients with severe OSA who demonstrated poor CPAP adherence. In this prospective interventional study, multilevel RFA was associated with a reduction in AHI from 86.03 to 54.65 events/hour, a decrease in required CPAP from 17.13 cm H_2_O to 10.97 cm H_2_O, and an increase in nightly CPAP use from 1.57 to 3.75 h [97]. While the study was limited by its relatively small sample size and lack of a control group, it introduced an important treatment paradigm by demonstrating that RFA may serve as an adjunctive therapy to improve CPAP tolerance rather than a standalone treatment for OSA. These findings have expanded the role of RFA in contemporary practice, particularly for patients with severe disease who struggle with adherence to PAP therapy despite ongoing treatment.

#### 6.5.3. Transoral Robotic Surgery (TORS)

Transoral robotic surgery (TORS) focuses on resecting tissue from the tongue base to decrease retrolingual obstruction that contributes to upper airway collapse during sleep. This procedure utilizes a da Vinci surgical robot to allow enhanced visualization of the airway and precision to avoid external excisions. Ideal candidates are those with a BMI < 25 kg/m^2^ with a rather large tongue base, specifically from lingual hypertrophy instead of lymphoid hyperplasia [98]. TORS has shown improvement in functional sleep scores as well as no significant loss of swallowing function [99]. Morbidity is generally low, with most series reporting no requirement for tracheostomy. The most common major complication is postoperative hemorrhage requiring intervention, occurring in approximately 5–8% of cases [100]. A 2021 systematic review and meta-analysis by Lechien et al. synthesized outcomes from 32 studies comprising more than 2500 patients undergoing TORS for OSA. Pooled analysis demonstrated a reduction in mean AHI from 44.3 to 17.8 events/hour, corresponding to an improvement of approximately 20–24 events/hour. Clinically meaningful improvements were also observed in patient-reported daytime sleepiness, with mean Epworth Sleepiness Scale scores decreasing from 12.3 to 5.8, indicating benefit in both objective disease severity and subjective daytime symptoms [101]. The large pooled patient population strengthened these findings in favor of TORS as a viable treatment option for carefully selected patients with tongue-base obstruction. However, the available literature remains limited by the predominance of observational studies, variability in surgical technique, and heterogeneity in patient selection.

### 6.6. Contemporary Multilevel Surgical Approaches

Single-stage minimally invasive surgery for OSA typically combines multiple procedures to improve airflow and oxygenation across several levels of obstruction. These approaches often address the nasal cavity (septoplasty, turbinate reduction), the palate (palatal implants, pharyngoplasty, UPPP), and the tongue base (RFA) [102]. By preserving the mucosa, these techniques are associated with lower postoperative morbidity compared to more invasive stand-alone procedures such as traditional UPPP [103]. The procedures are also relatively low intensity and can frequently be performed in an office-based setting rather than the operating room.

Multilevel surgical strategies are used to target both retropalatal and retrolingual obstruction in a single treatment plan. Modern surgical management of SRBD, particularly OSA, increasingly relies on multilevel treatment algorithms rather than an isolated single-site procedure. Upper airway obstruction frequently occurs at multiple levels, including the velum, lateral pharyngeal walls, tongue base, and epiglottis [32]. As a result, treatment strategies have evolved beyond traditional staged approaches toward multilevel interventions. Contemporary surgical algorithms emphasize patient-specific treatment selection rather than a uniform procedural approach. Surgical planning typically begins with confirmation of PAP intolerance or failure, followed by comprehensive upper airway evaluation incorporating physical examination, craniofacial assessment, imaging, and DISE [104].

Findings are then used to characterize the primary sites and patterns of collapse. In addition, physiologic phenotyping may further refine operative planning and facilitate individualized treatment selection. For example, isolated retropalatal or lateral pharyngeal wall collapse may be managed with palatal procedures such as expansion sphincter pharyngoplasty or anterior palatoplasty, whereas predominant tongue-base obstruction may warrant tongue-base reduction, genioglossus advancement, or HNS [104]. Skeletal deficiencies, including maxillomandibular hypoplasia, may favor maxillomandibular advancement [104]. When obstruction is identified at multiple airway levels, combined multilevel procedures are often recommended. This algorithmic approach has shifted sleep surgery away from traditional single-site operations toward individualized interventions tailored to each patient’s unique anatomic and physiologic profile to maximize efficacy while avoiding unnecessary procedures. While these approaches are most established in OSA, similar principles may be applied in selected patients with other SRBDs characterized by upper airway obstruction, such as upper airway resistance syndrome or syndromic and craniofacial disorders [29].

The SAMS randomized controlled trial showed that multilevel airway surgeries can significantly improve both AHI and patients’ reported daytime sleepiness in appropriately selected patients with moderate-to-severe OSA [32]. In this trial, multilevel interventions, including UPPP combined with tongue-base reduction, RFA, or midline glossectomy tailored to the patient’s pattern of airway collapse, significantly improved OSA severity. These interventions also produced clinically meaningful improvements in daytime sleepiness, with Epworth Sleepiness Scale scores decreasing by more than 3 points. Serious adverse event rates were similar to those observed with UPPP alone, and no participants reported significant long-term functional difficulties. This integrated approach supports a more targeted selection of surgical interventions based on the specific pattern of airway collapse identified in each patient.

Although DISE was initially developed to identify surgical candidates, it has increasingly been used to guide individualized surgical planning. Comparing surgical success rates with and without DISE demonstrates a marked improvement in surgical success rates (defined above), with 86% of patients achieving success with DISE vs. 51.4% without [59]. When compared specifically in use with single-stage multilevel surgery, success rates range from 65 to 82. A 2026 cohort study evaluating expansion sphincter pharyngoplasty, anterior palatoplasty, and tongue base resection reported a 65% surgical success rate, with 45% of patients achieving an AHI < 5 events/hour [105]. Similarly, a 2021 study of 24 patients undergoing multilevel surgery incorporating expansion sphincter pharyngoplasty, coblation tongue base reduction, and partial epiglottectomy demonstrated an 82.3% success rate, with AHI improving from 33.0 to 17.7 events/hour and Epworth Sleepiness Scale scores improving from 11.0 to 7.9 (*p* < 0.05) [106]. Although these studies are limited by their observational design and relatively small patient populations, they provide practical evidence supporting the use of DISE to guide individualized multilevel surgical planning. Collectively, these findings suggest that patients with complex patterns of airway collapse may benefit from tailored multilevel interventions rather than isolated procedures directed at a single anatomic site.

Overall, contemporary multilevel surgical strategies guided by DISE and patient-specific treatment algorithms offer a personalized approach to SRBD management, with evidence demonstrating meaningful improvements in both objective and patient-reported outcomes.

### 6.7. Surgical Approaches in Other SRBDs

Traditional upper airway surgeries, including UPPP, may similarly influence respiratory physiology beyond simple enlargement of the airway lumen. By reducing sites of pharyngeal collapse and decreasing obstructive event frequency, these procedures may lessen intermittent hypoxia, excessive respiratory effort, and repeated arousals that can destabilize ventilatory control. However, because CSA is primarily driven by abnormalities in respiratory control rather than fixed obstruction, improvement in central events is expected to be variable and dependent on the underlying physiologic phenotype.

## 7. New Technologies in the Management of SRBD

### 7.1. Emerging Surgical Techniques

Advances in diagnostic technologies have been mirrored by rapid innovation in the management of SRBDs, with a growing emphasis on precision, personalization, and minimally invasive approaches. Emerging surgical techniques and device-based therapies increasingly target specific anatomic and physiologic contributors to collapse. At the same time, technological improvements in PAP therapy and implantable devices have enhanced treatment efficacy, comfort, and adherence. These developments reflect a shift toward more tailored, patient-centered management strategies in SRBDs.

#### 7.1.1. Virtual Surgical Planning

Virtual surgical planning (VSP) has been evaluated as an adjunct to maximize precision for MMA surgeries using computer-aided design. This technology allows surgeons to create a three-dimensional model of the patient’s anatomic features to pre-plan surgical interventions and minimize complications. The three-dimensional (3D) models of the dental–skeletal anatomy before surgery as well as allowing for proposed mechanisms for alignment during surgery.

In a 2025 prospective study evaluating VSP-guided orthognathic surgery in patients with OSA, more than 50% reduction in AHI was observed, resulting in a reported surgical success rate of 100% [107]. Beyond clinical outcomes, the study demonstrated high accuracy between planned and achieved skeletal movements, supporting the feasibility of incorporating virtual planning into surgical workflows. While the study population was relatively small, the findings highlight the potential role of VSP in improving surgical precision and optimizing patient-specific treatment planning. Future studies involving larger patient populations and longer follow-up periods will help determine the reproducibility of these results across broader clinical settings. A further limitation of VSP is that current models may incompletely account for dynamic changes in facial structure, potentially affecting the accuracy of predicted postoperative outcomes.

#### 7.1.2. Hypoglossal Nerve Stimulation

The hypoglossal nerve plays a central role in maintaining pharyngeal patency by activating the genioglossus and other dilator muscles [108]. During sleep onset, hypoglossal nerve output gradually decreases, resulting in reduced upper airway muscle tone. This reduction increases airway resistance and may lead to narrowing or complete collapse of the retrolingual airway. To counteract this mechanism, an implantable device was developed to stimulate the hypoglossal nerve. These systems deliver electrical stimulation to the hypoglossal nerve via a cuff electrode, causing contraction of the genioglossus muscle either with inspiration or continuously. Clinical studies have demonstrated significant reductions in AHI, with decreases of about 68% observed 12 months after implantation [109]. Hypoglossal Nerve Stimulation (HNS) has emerged as a successful, FDA-approved, second-line surgical therapy for OSA patients who cannot tolerate PAP. It has high reported success rates and a targeted mechanism of action, which addresses retrolingual collapse. HNS may be particularly effective in patients with impaired upper airway muscle responsiveness, one of the principal endotypic traits within the PALM framework.

New models are being developed yearly, with a model being released in 2025 that uses a battery-free implant instead of surgical implantation. Studies are ongoing for a model that places an electrode via ultrasound guidance with similar outcomes [110]. Although most long-term outcome data derive from the Inspire system, including the STAR trial, recent technological developments have expanded the hypoglossal stimulation landscape. In 2025, the FDA approved the bilateral Genio system, which utilizes a leadless, battery-free design and bilateral hypoglossal nerve stimulation, potentially broadening treatment options for appropriately selected patients [111]. Another major innovation is proximal hypoglossal nerve stimulation. This approach utilizes a multicontact electrode array to selectively stimulate sectors of the proximal nerve rather than the distal trunk. By enabling more targeted stimulation, this technology may simplify implantation and expand patient eligibility by reducing reliance on DISE-based selection criteria. The OSPREY randomized controlled trial evaluated proximal hypoglossal nerve stimulation in patients with moderate-to-severe OSA. This study demonstrated that 58.2% of treated patients achieved the primary endpoint, defined as a >50% reduction in AHI with a post-treatment AHI < 20 events/hour, compared with 13.5% of controls [112]. Additional innovations, including MRI-compatible systems and less invasive implantation techniques, continue to evolve and may further expand the role of HNS in OSA management.

Current candidates for hypoglossal nerve stimulation are typically adults with moderate-to-severe OSA who are unable to tolerate CPAP therapy. Criteria for hypoglossal nerve stimulation initially required an AHI of 15–65 events/hour and a BMI ≤ 32 kg/m^2^. In 2023, the FDA expanded eligibility to patients with BMI ≤ 40 kg/m^2^ [113]. Additional requirements include documented CPAP intolerance, absence of central or mixed apnea comprising more than 25% of total AHI, and absence of complete concentric collapse (CCC) of the soft palate on DISE [114].

Beyond excluding CCC, DISE has become an important tool for identifying factors associated with treatment success. In a multicenter study of 343 patients, complete oropharyngeal lateral wall (OLW) collapse was associated with lower surgical response rates (58% vs. 74%), whereas complete tongue-related obstruction was associated with improved outcomes (78% vs. 68%) [115]. The multicenter design and large patient population strengthened the generalizability of these findings and helped establish DISE as a valuable adjunct for refining patient selection rather than simply determining eligibility. Similar findings were seen in a prospective study of 369 patients, which showed that OLW collapse reduced HNS treatment success by 18% compared to patients without OLW collapse. The study also found that airflow shape analysis from home sleep testing could predict the likelihood of OLW collapse, raising the possibility of identifying poor HNS candidates without the need for DISE in select patients [116]. Additional support for the predictive value of DISE comes from a study demonstrating that patients with complete baseline collapse of both the velum and lateral walls experienced significantly smaller reductions in AHI following HNS compared to those with partial collapse (4.4 vs. 22.3 events/hour) [117]. Collectively, these studies have shifted the role of DISE from a simple exclusionary test toward a tool that can better predict treatment response and facilitate individualized patient selection. However, as previously stated, consideration remains regarding the interobserver variability of DISE during such preoperative decisions.

Long-term data support the durability of HNS therapy. The landmark STAR trial prospectively evaluated upper airway stimulation in patients with moderate-to-severe OSA who were intolerant of CPAP [109]. They demonstrated a reduction in median AHI from 29.3 to 9.0 events/hour, with benefits maintained at 5 years and a surgical success rate of 75% at 60 months [109]. As a prospective multicenter trial with extended follow-up, STAR provided some of the earliest high-quality evidence supporting the long-term efficacy of HNS and was instrumental in establishing the therapy as a viable alternative for appropriately selected CPAP-intolerant patients. A 2020 systematic review and meta-analysis by Costantino et al., incorporating 12 studies and 350 patients, demonstrated sustained improvements in AHI, oxygen desaturation index, and Epworth Sleepiness Scale scores across extended follow-up periods [118]. This further supported the benefits observed in STAR were reproducible outside of a single trial setting. Similar results have been observed in real-world practice through the ADHERE registry, which enrolled more than 1000 patients across multiple centers and demonstrated that approximately 76% of patients achieved an AHI below 15 events/hour following implantation [114]. The consistency of outcomes across prospective trials, meta-analyses, and large registry studies has provided numerous pieces of evidence supporting the long-term effectiveness of HNS and its emergence as a standard treatment option for appropriately selected patients with CPAP-intolerant OSA.

Collectively, these findings have established HNS as a durable and increasingly personalized treatment option for CPAP-intolerant OSA, with growing evidence demonstrating that careful patient selection through DISE and other predictive tools can optimize outcomes and further expand its role in contemporary sleep surgery.

### 7.2. Technological Advancements in CPAP

Advancements in PAP technology have dramatically improved treatment accessibility, patient comfort, and adherence in the management of SRBD. Remote monitoring has also advanced the management of PAP therapy. Real-time transmission of data, including residual AHI, airflow leaks, and pressure requirements, is now available to clinicians [119]. This information facilitates earlier recognition of issues with PAP therapy, promoting earlier interventions or adjustments of therapy to optimize treatment. Remote monitoring is also available through telemedicine services, improving access to specialty services while reducing the need for in-person follow-up visits.

Technological innovations in the CPAP interface have also been introduced to improve tolerance and adherence. Mask discomfort and air leak are common barriers to effective PAP use. Compared with traditional full-face masks, nasal masks and nasal pillows have been associated with improved comfort, reduced claustrophobia, and greater adherence [42]. Additionally, using lighter materials for masks, improving seal mechanisms, and customizing the mask fit have improved patient satisfaction. Heated humidification represents another important adjunct to PAP therapy, reducing mucosal dryness and upper airway irritation.

#### Advancement in PAP Modalities

One of the most significant developments is the APAP device, which continuously monitors airflow dynamics and automatically adjusts pressure on a breath-to-breath basis throughout the night. One major advantage of APAP devices is that they do not require a titration study to find the optimal pressure setting like earlier CPAP devices [119]. The ability to initiate PAP therapy without requiring a patient to stay overnight for in-person laboratory titration improves compliance and is more cost-effective. A 2019 American Academy of Sleep Medicine (AASM) systematic review, meta-analysis, and GRADE assessment synthesized evidence from numerous randomized controlled trials comparing APAP and CPAP therapy. The review found no clinically significant differences in treatment efficacy between the two modalities for OSA, while demonstrating modest improvements in adherence with APAP devices [42]. The methodology, inclusion of randomized studies, and formal GRADE assessment allow for a tangible and applicable alternative that can be incorporated into contemporary clinical practice. Current guidelines support initiation of PAP therapy using either home-based APAP or in-laboratory titration in appropriately selected patients with uncomplicated OSA. However, in-laboratory titration remains important for patients with more complex forms of SRBD or significant comorbidities. Although APAP is generally not appropriate for patients with CSA or hypoventilation syndromes.

BiPAP was developed to better approximate physiologic breathing patterns. In contrast to CPAP, which delivers a constant pressure throughout the respiratory cycle, BiPAP provides higher inspiratory positive airway pressure (IPAP) and lower expiratory positive airway pressure (EPAP), thereby reducing the work of breathing for patients. The same AASM review found that BiPAP does not routinely demonstrate superior efficacy compared with CPAP in patients with uncomplicated OSA [42]. Given the lack of demonstrated superiority over CPAP, BiPAP is not routinely recommended as first-line therapy for uncomplicated OSA and is generally reserved for selected patients who are intolerant of CPAP or require alternative pressure delivery strategies.

**Figure 1:**
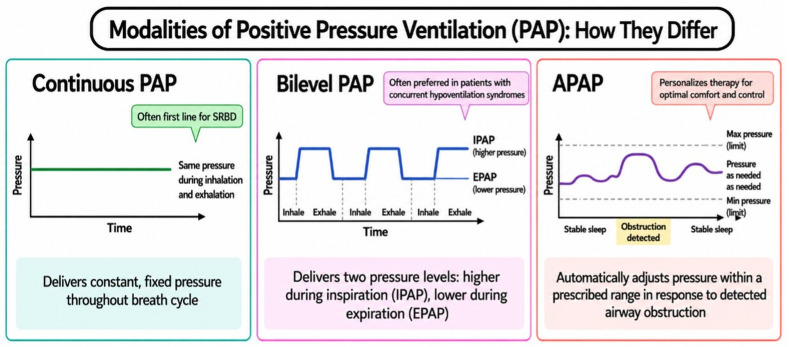
Graphical representation of PAP modalities showing pressure changes over time. CPAP, which provides continuous pressure throughout (**left**); BiPAP, which offers differing force during inspiration and expiration (**middle**); and APAP, which automatically adjusts based on detected pressures (**right**).

An important advancement in this modality is auto–bilevel positive airway pressure (Auto-BiPAP), which dynamically adjusts IPAP and EPAP in real time according to patient needs [120]. In a prospective study of patients transitioning from CPAP to Auto-BiPAP, there was a significant reduction in expiratory pressure (from 12 to 8 cmH_2_O), 95th percentile pressure, and mask leak. These physiologic improvements were accompanied by better Pittsburgh Sleep Quality Index scores and resolution of CPAP-related adverse effects, including xerostomia, choking sensation, and aerophagia, with 90% of patients ultimately preferring BiPAP over CPAP [121]. The prospective design allowed for assessment of both objective treatment parameters and patient-reported outcomes, highlighting that treatment success extends beyond reductions in respiratory events alone. While the study was limited by its relatively small sample size and lack of a parallel control group, the consistent improvements in comfort, sleep quality, and patient preference support the role of Auto-BiPAP as an important option for patients who struggle with CPAP-related side effects despite adequate treatment efficacy. A comparison of PAP modalities is shown in Figure 1 and Table 3.

Further advancing noninvasive ventilation, volume-assured pressure support (VAPS) devices automatically adjust IPAP to maintain a preset target tidal volume or minute ventilation. These systems also modulate respiratory rate and auto-EPAP to address both hypoventilation and upper airway obstruction. BiPAP with a backup rate (BiPAP S/T) and VAPS are particularly beneficial in sleep-related hypoventilation disorders, such as obesity hypoventilation syndrome, restrictive lung disease, and neuromuscular conditions, where additional ventilatory support is needed to improve carbon dioxide clearance [122]. BiPAP is also a reasonable alternative for patients needing therapeutic pressures over 20 cm H_2_O, the maximum for CPAP devices.

Adaptive servo-ventilation (ASV) provides breath-by-breath adjustment of inspiratory pressure support. These devices will continuously measure minute ventilation or airflow to calculate a target ventilation level, after which the machine will adjust its pressure depending on readings [123]. It will increase inspiratory support during hypopnea, withdraw support during hyperventilation, and provide mandatory breaths during apnea. ASV is highly indicated in select patient populations, including patients with Cheyne–Stokes respiration and treatment-emergent complex sleep apneas. In the Complex Sleep Apnea Resolution Study, a prospective randomized controlled trial comparing CPAP and ASV, patients treated with ASV experienced significantly greater reductions in residual apnea burden and were more likely to achieve normalization of their apnea–hypopnea index than those treated with CPAP alone. These results helped establish ASV as a preferred treatment option for patients with persistent central respiratory events despite CPAP therapy [124]. Additional evidence has expanded the role of ASV in opioid-induced CSA. In a prospective study, ASV demonstrated that CPAP failed to adequately control central respiratory events, with a residual central apnea index of approximately 20 events/hour. Following transition to ASV, the central apnea index decreased to 0 events/hour, oxygen saturation improved, and long-term adherence averaged 5.1 ± 2.5 h per night over follow-up periods extending up to 6 years [125]. While the evidence base for opioid-induced CSA remains smaller than that for treatment-emergent CSA, these findings support consideration of ASV in carefully selected patients when conventional PAP modalities prove ineffective.

In select patients, ASV has shown significantly more effectiveness than the other pathways, such as CPAP or BiPAP. When comparing treatment rates in complicated sleep apnea, a randomized controlled trial showed ASV achieved 89.7% success (AHI < 10 events/hour) at 90 days versus 64.5% with CPAP (*p* = 0.0214), with a mean AHI of 4.4 versus 9.9 events/hour, respectively [126].

One attractive outcome of SRBD therapy targets the downstream consequence of sleep apnea, particularly its natural course towards the possible development and progression of heart failure with reduced ejection fraction (HFrEF). However, evidence supporting ASV for this purpose remains limited. The ADVENT-HF trial randomized 731 patients with HFrEF and severe OSA or CSA to receive ASV plus usual care versus usual care alone [127]. In contrast to the earlier SERVE-HF trial, which primarily enrolled patients with CSA, ADVENT-HF included a predominantly OSA population and largely nonsleepy individuals. Despite effectively reducing sleep-disordered breathing, ASV did not significantly reduce the primary composite endpoint of all-cause mortality, cardiovascular hospitalization, new-onset atrial fibrillation, or appropriate ICD shock, nor did it improve all-cause mortality [127]. The large randomized design and clinically meaningful cardiovascular endpoints strengthen the applicability of these findings and suggest that improvements in respiratory parameters do not necessarily translate into improved cardiovascular outcomes. However, significant improvements were observed in sleep quality and overall quality of life, highlighting the potential symptomatic benefits of therapy even in the absence of measurable cardiovascular benefit. A limitation of the study is that the CSA subgroup was relatively small, making it difficult to determine whether patients with OSA and CSA respond differently to ASV. Future studies focusing on individual sleep apnea phenotypes may help clarify whether certain patient populations derive greater cardiovascular benefit from therapy. These findings have shifted expectations regarding ASV in HFrEF, supporting its role in symptom management rather than as a strategy to reduce cardiovascular morbidity or mortality.

### 7.3. Transvenous Neurostimulation

Another device outside of PAP therapy is a transvenous neurostimulation, used specifically for CSA with concurrent heart failure, approved by the FDA in 2017. This implantable system delivers transvenous stimulation to the phrenic nerve, resulting in diaphragmatic contraction that closely mimics physiologic breathing [128]. In a multicenter randomized controlled trial, Costanzo et al. demonstrated that more than 50% of patients treated with phrenic nerve stimulation achieved a reduction in AHI of at least 50% at 6 months, accompanied by significant improvements in oxygenation and sleep-related outcomes. Importantly, 91% of patients remained free from serious device-related adverse events, supporting a favorable safety profile [128]. Although longer-term data continue to emerge, this study established transvenous phrenic nerve stimulation as a promising treatment option for carefully selected patients with CSA, particularly those who remain symptomatic despite conventional therapies or are unable to tolerate PAP-based treatment.

## 8. Personalized Approaches in SRBD Treatment

### 8.1. Molecular Mechanisms and Personalized Treatment Strategies in SRBD

Clinical phenotypes further complement endotype-based assessment by grouping patients according to observable characteristics such as obesity, excessive daytime sleepiness, cardiovascular risk burden, craniofacial anatomy, and symptom profiles. Advances in the understanding of SRBD have revealed that these conditions are associated with important metabolic, vascular, and neuropsychiatric consequences. Patients with similar AHI values may demonstrate markedly different symptoms, comorbidity burdens, and responses to therapy. For example, individuals with obesity-related OSA may benefit substantially from GLP-1 receptor agonists and bariatric surgery, whereas patients with predominant craniofacial abnormalities may be better candidates for skeletal reconstruction procedures. Integrating both phenotypic and endotypic information allows clinicians to select therapies that address the underlying mechanism of disease rather than relying solely on disease severity classifications. This approach forms the foundation of precision medicine and individualization of management in SRBD. Identification of these molecular pathways has improved understanding of disease pathogenesis while simultaneously revealing potential therapeutic targets and opportunities for more individualized treatment strategies.

#### 8.1.1. Obesity

One of the main risk factors for OSA remains obesity; studies have been conducted linking genetic factors in obesity to OSA. Peroxisome proliferator-activated receptor gamma (PPAR-ɣ) has been a focus of obesity-centered studies. The proposed mechanism involves activated macrophages in adipose tissue contributing to the pathogenesis of obesity and other complications, such as insulin resistance and inflammation. PPAR-ɣ helps regulate the inflammation caused by macrophages. However, in multiple lung pathologies such as adult respiratory distress syndrome, there are notable PPAR-ɣ deficiencies, suggesting that PPAR-ɣ has a role in the pulmonary system regulation. Studies on patients with OSA without underlying lung pathology have similarly demonstrated a notable decrease in alveolar macrophages’ PPAR-ɣ functional activity and expression [129]. This highlights a potential mechanism linking SRBD and metabolic dysfunction.

Leptin dysregulation provides another connection to obesity and OSA [130]. Leptin is a peptide hormone that promotes appetite control and boosts energy expenditure, and when resistance develops, it disrupts normal calorie regulation. Chronic intermittent hypoxia (CIH) is a physiologic stress marker associated with sleep disturbances from OSA. Studies found that after approximately 3.5 months of frequent CIH events, there was significantly increased circulating leptin as well as progressive leptin resistance, which is followed by weight gain, hyperglycemia, and increased oral intake. As soon as 2 weeks of CIH events led to increased leptin levels compared to patients without OSA [130]. These findings suggest that OSA may worsen existing obesity and related syndromes and contribute to the development of obesity in non-overweight patients with OSA.

Researchers obtained gene expression microarrays of patients’ adipose tissue in patients with and without OSA. They isolated 25 total differentially expressed genes (DEGs) related to obesity: 13 genes were upregulated, and 12 were downregulated [131]. Machine-learning models highlighted XRCC4 and ARL6 as promising biomarkers within the DEGs for distinguishing OSA from non-OSA patients. XRCC4’s proposed mechanism of action included DNA damage from the chronic inflammatory state of obesity, while ARL6 was thought to intervene in the function of adipose and signaling pathways [131]. However, further studies are required to fully understand the mechanisms of these genetic markers.

Obesity remains one of the most important modifiable risk factors for OSA and a major therapeutic target in disease management. Excess adipose tissue contributes to upper airway narrowing, while obesity-related metabolic dysfunction and systemic inflammation worsen disease severity. Consistent with this relationship, a 2024 meta-analysis demonstrated that a 20% reduction in BMI was associated with a 57% reduction in OSA severity as measured by AHI, while longitudinal studies have shown that a 10% reduction in body weight correlates with a 26% decrease in AHI [37]. These findings suggest that targeting obesity can reduce OSA severity rather than just improve symptoms. Consequently, advances in obesity treatment have generated significant interest in metabolic therapies [132]. As discussed previously, tirzepatide became the first FDA-approved medication for moderate-to-severe OSA in adults following the SURMOUNT-OSA trial, where its treatment reduced AHI by 58.7% from baseline while also improving hypoxic burden, body weight, and patient-reported sleep impairment [48]. The growing understanding of obesity-related pathways, including leptin dysregulation and metabolic dysfunction, has helped establish obesity itself as a therapeutic target rather than simply a risk factor for OSA.

#### 8.1.2. Endothelial Dysfunction and Cardiovascular Disease

Endothelial dysfunction has been evaluated as a contributor to the higher risk of atherosclerotic disease in OSA. Overall, endothelial dysfunction holds a high correlation with coronary events such as myocardial infarction or acute coronary syndrome [133]. The degree of endothelial vasomotor regulation can be assessed by measuring nitric oxide (NO), a critical vasodilator. Patients with OSA exhibited a reduced level of circulating NO compared with that of patients without OSA, suggesting that OSA can induce a chronic inflammatory state [133]. However, with adequate treatment, such as CPAP or surgical intervention, normalization of NO levels can occur [134].

Additionally, as a result of recurrent episodes of hypoxemia during the sleep cycle, it is proposed that there is ongoing vascular injury. The recovery process leads to the release of ROS, which perpetuates vascular injury and inflammation. Down cycle, this can further lead to decreased NO production and leave the patient at increased risk of other vascular conditions [135]. Levels of C40 ligand, which can be used as a surrogate marker for the presence of atherosclerotic disease, have been proven to be elevated in patients with OSA regardless of their other risk factors [136]. This perspective remains critical when caring for patients with OSA to reinforce the need for treatment to decrease the risk of atherosclerotic disease.

Early studies are investigating the use of stem cells to protect and repair cells from inflammation, endothelial stress, and ROS, which are thought to contribute to the worsening of OSA. Available data is scarce; however, it remains a topic of interest, and stem cell research continues to evolve.

Given the concurrent elevated risk of cardiovascular and atherosclerotic disease, studies have been conducted to highlight the importance of the incorporation of physical activity into patients’ routine in addition to CPAP therapy [137]. Researchers selected intervention mapping (IM) as a technique to help providers assess overall health risk and identify barriers to physical activity. Using this information, providers then developed strategies to meet predetermined objectives while also addressing the behavioral changes needed to achieve those goals via the IM technique. Although the study primarily focused on intervention design rather than clinical outcomes, it highlights growing interest in integrating behavioral and lifestyle interventions into comprehensive OSA management.

#### 8.1.3. Neuropsychiatric Consequences of OSA

A large prospective cohort study found that patients diagnosed with OSA demonstrated a statistically significant increase of approximately 39–44% in adverse mental health outcomes, including depressive symptoms, distress, diagnosis of a mental health disorder, or antidepressant use. The likelihood of these outcomes increased progressively with greater OSA risk, with depressive symptoms showing the strongest association [138]. However, the observational nature of the study inherently does not draw conclusions on causation and should thus be interpreted with caution.

For example, studies found that there is an increased prevalence of OSA in patients with major depressive disorder and post-traumatic stress disorder (PTSD). Among veterans with comorbid OSA and PTSD, PAP therapy has been associated with a significant reduction in PTSD symptom severity, depression scores, nightmare frequency, and daytime sleepiness. Conversely, PTSD has been linked to reduced PAP adherence; however, greater PAP adherence has been associated with larger improvements in PTSD symptoms [139]. This only highlights the complex interaction between psychiatric diseases and OSA management, emphasizing the importance of recognizing and addressing both conditions to optimize treatment outcomes.

#### 8.1.4. Surgical Treatment Approaches Based on Phenotypic SRBD Presentation

With largely varying presentations of SRBD, decisions on appropriate management are best tailored to each patient and their presenting phenotype. In populations whose SRBDs are predominantly secondary to anatomical features, surgical management is the obvious path. However, nuances arise when anatomical variants are not as clear-cut and may only manifest through physiologic changes measured through diagnostic testing. As previously described, the PALM framework provides a basis for individualized treatment, particularly in choosing the optimal surgical approach.

Growing recognition of overlapping mechanisms has led to a shift toward classification systems that go beyond the traditional OSA versus CSA framework. Rather than classifying a patient solely by the presence of airway obstruction, a mechanism-based approach incorporating traits such as airway collapsibility, ventilatory control stability, arousal threshold, and muscle responsiveness may better target treatment response. Emerging AI-based classification systems increasingly demonstrate that sleep apnea exists along a continuous spectrum rather than binary OSA vs. CSA categories. As such, surgical interventions should not be interpreted as exclusively anatomic therapies but rather as interventions capable of modifying broader sleep-respiratory dynamics in select patients.

Reductions in obstructive respiratory events and improvements in oxygenation following targeted interventions may attenuate intermittent hypoxia-mediated chemoreflex activation and reduce ventilatory instability in susceptible patients [140]. For example, MADs may decrease upper airway collapsibility by advancing the mandible and increasing pharyngeal airway volume, which can reduce obstructive events and associated fluctuations in oxygen and carbon dioxide levels. By minimizing recurrent respiratory disturbances and arousals, MAD therapy may indirectly improve ventilatory control stability in patients with mixed obstructive and central features. Similarly, MMA produces a more permanent increase in upper airway dimensions and has been associated with substantial reductions in obstructive burden, potentially decreasing physiologic stressors that contribute to ventilatory instability [85].

Neuromodulatory therapies such as HNS primarily target upper airway muscle dysfunction. However, they may also indirectly improve ventilatory stability by reducing sleep fragmentation and promoting more consistent respiratory timing through improved afferent feedback and reduced loop gain variability [109]. By restoring coordinated activation of the genioglossus during inspiration, HNS may decrease repetitive airway collapse and reduce the arousal-driven fluctuations in respiratory effort that contribute to unstable breathing patterns. Although HNS is not a treatment for CSA, its ability to stabilize upper airway mechanics may influence downstream ventilatory control in selected patients with mixed obstructive and central events [109]. Similarly, weight-loss interventions may improve both upper airway anatomy and physiologic factors that contribute to ventilatory instability. While these therapies should not be considered primary treatments for CSA, they illustrate how interventions traditionally viewed as exclusively anatomic may exert broader effects on sleep-respiratory physiology.

### 8.2. Expansion of Artificial Intelligence

With the exponential growth of AI use in medicine, AI is expected to become an integral part of sleep medicine that will support faster diagnosis. With integration into reading patient sensor data, real-time information can be provided to adjust the therapy strategy. Current reviews show 98.8% accuracy with 99.1% sensitivity and 98.5% specificity for AI models being able to classify OSA and CSA [141]. As this field is further expanded, it may even lead to patients being able to screen themselves at home.

### 8.3. Patient-Centered Decision Making

Patient-centered decision making (PCDM) remains a cornerstone of delivering high-quality care, recognizing that patient preferences and treatment goals contribute to adherence and outcomes. The cornerstone of PCDM remains involving the patient in shared decision-making, offering them relevant clinical data and options for treatment plans that align with their priorities.

A focus of studies has been creating a patient-centered sleep study report (PCSR) to optimize patients’ understanding of their disease and empower them to participate in decision-making [142]. The PCSR involved results from polysomnography reporting that included AHI, oxygen desaturation index, and arousal index in simple terminology; also included were simple explanations of treatment options. The implementation of this technique led to improved patient understanding, and the long-term effects on compliance remain under study.

An alternative decision aid has been developed, Decide2Rest, to guide PCDM in older adults above age 60 with OSA. The Decide2Rest similarly included general information about OSA, their treatment results, as well as explanations of different treatment options with risk-benefit analysis [143]. When compared to a control group without the intervention, those in the Decide2Rest program felt more informed about treatment options and prepared to make decisions [143].

Together, these findings support integrating PCDM and shared decision-making tools into SRBD treatment to ensure evidence-based choices that reflect patient values and improve engagement and long-term outcomes. Key features to highlight include providing the patient with information about their disease and severity, as well as varying treatment options so they can make an informed decision.

### 8.4. Economic Considerations of SRBD on Patients

Economic factors constrain the adoption of new technologies in regular practice. Even with the current standard therapy, CPAP, the cost creates a financial barrier to care for patients with poor socioeconomic status. Given that these new devices are novel and not yet standard of care, there is not yet a clear reimbursement pathway that can offload the cost onto patients. In a similar pattern, device affordability and the need for specialized diagnostic services disproportionately leave patients in underserved communities or low-resource regions unable to access this care [144]. It remains difficult for uninsured patients to be offered guideline-based therapy.

Notably, throughout the United States, there is a shortage of sleep medicine physicians who would serve as the primary means for communicating these innovations to patients and other providers [145]. Currently, there is a lack of formal training in fellowship programs for telemedicine or remote work in sleep medicine. As more advances in sleep medicine move toward home-based testing, this widens the gap between patients receiving care. Telemedicine-based sleep programs have improved access, but challenges with workflow changes, provider training, and patient onboarding show that even reliable technologies require considerable operational work before they fit seamlessly into routine practice.

## 9. Conclusions

Advancements in diagnostic modalities, surgical techniques, and device-based therapies are reshaping the management of SRBD, moving toward individualized care. The future of SRBD includes a range of innovative technologies, from diagnostic measurements and modalities to advances in treatment approaches.

Diagnostic evaluation has expanded beyond laboratory polysomnography into the home setting, with innovations in remote testing strategies improving accessibility. In addition to polysomnography, tools such as PAT and wireless sleep sensors are becoming more widespread in use. As more clinical data on relevance and indications become clear, we anticipate that these methods will shape the landscape of SRBD diagnosis in the near future. These monitoring devices allow for increased accessibility and can facilitate improved patient–clinical interaction, automated data sharing from home devices, and early detection of compliance lapses, all of which lead to improvement in clinical outcomes. OCT also provides additional information and serves as an innovative diagnostic tool, although its clinical utility remains incompletely established and is best viewed as an adjunct to existing diagnostic modalities.

Advances in diagnostic measurements beyond AHI are also promising, including hypoxic burden, ODI, T90, arousal index, and sleep structure. The physiologic basis of each measurement enhances the physiologic understanding of SRBD, both on a population and individual level. These measurements, along with the PALM framework, are becoming more established, with growing research interest, and will likely be the new standard for categorizing and subsequently managing SRBD patients in the near future.

All of these advancements prime the patient for the optimal treatment, which has shifted focus to hybrid and/or individualized approaches to best manage each patient. Newer studies support the use of multiple modalities across different methodologies, incorporating core non-invasive therapies with adjunctive treatments such as surgical modifications. Additionally, individualized surgical approaches are emerging as promising methods for SRBD management.

From diagnosis to treatment and assessment, innovations within the field of SRBD are far from limited. With the ongoing advancements in AI technology as well as virtual surgical planning, there are promising opportunities to further personalize diagnosis and treatment. As diagnostic and therapeutic technologies evolve, the future of SRBD management will tailor interventions to the patient’s unique phenotypic profile by following a comprehensive, patient-centered, and multi-modality approach toward patient care.

## Figures and Tables

**Table 1:** Comparison of Current and Emerging Diagnostic Approaches for Sleep-Related Breathing Disorders.

Feature	Polysomnography (PSG)	Peripheral Arterial Tonometry (PAT)	Wireless Sleep Sensors
Clinical Status	Gold-standard diagnostic test	FDA-approved home sleep testing modality	Investigational technology
Testing Environment	Sleep laboratory	Patient’s home	Patient’s home
Physiologic Signals Measured	EEG, EOG, EMG, airflow, respiratory effort, oxygen saturation, ECG	Peripheral arterial tone, pulse oximetry, and actigraphy	EEG, EOG, EMG via wireless forehead and chin sensors
Diagnostic Accuracy	Reference standard	Best performance in moderate-to-severe OSA; variable performance in mild disease	Under active validation against PSG
Major Advantages	Comprehensive physiologic assessment; definitive diagnosis	Convenient, accessible, no laboratory titration required	Potential for comprehensive physiologic monitoring in the home environment
Major Limitations	Expensive, labor-intensive, and limited accessibility	May overestimate disease severity; significant misclassification in mild-to-moderate OSA; abnormal results often require confirmatory PSG	Not yet commercially available; accuracy and long-term reliability remain under investigation
Most Appropriate Use	Definitive diagnosis and complex cases	Patients with a high pretest probability of OSA	Future large-scale home-based sleep monitoring

**Table 2:** Comparison of surgical techniques listed by modality, examining key outcomes, advantages, and limitations.

Modality		Mechanism	Key Outcomes	Advantages	Limitations	Precision Medicine Considerations
Uvulopalatopharyngoplasty (UPPP)	UPPP Alone	Resection and reshaping of the uvula, soft palate, and oropharyngeal soft tissue to enlarge the retropalatal airway	Cure (AHI < 5): ~24% AHI < 10: ~33% at 6 months	Most established palatal procedure; widely performed; can improve airflow in selected patients	Highly variable success; limited efficacy in multilevel collapse; outcomes depend on a strict definition of “success”	Ideal for patients with predominant retropalatal obstruction; less effective in multilevel disease or significant tongue base collapse
	UPPP + Tonsillectomy	Palatal expansion with removal of tonsillar obstruction improves retropalatal patency	~60% AHI reduction vs. 11% control; mean AHI 53.3 → 21.1 events/hour	Better outcomes in tonsillar hypertrophy; improved response vs. isolated UPPP	Limited generalizability (younger, lower BMI cohorts); residual moderate OSA common	Ideal for patients with tonsillar hypertrophy with favorable palatal anatomy
Lateral expansion pharyngoplasty (LEP)		Repositioning of pharyngeal musculature to increase lateral wall tension and stability	Improved airway stability and sleep outcomes (no standardized pooled AHI)	Targets lateral wall collapse; more physiologic than resection-based UPPP	Postoperative dysphagia; limited adoption	Ideal for patients who demonstrate DISE with lateral pharyngeal wall collapse
Expansion sphincter pharyngoplasty (ESP)		Isolation and anterior-superior rotation of the palatopharyngeus to stiffen the lateral pharyngeal wall	Improved lateral wall collapse; reduction in OSA severity in selected patients	Better control of lateral wall collapse vs. UPPP	Technical complexity; variable outcomes; still procedure-dependent	Ideal for patients who demonstrate DISE lateral wall collapse and poor response expected from traditional UPPP
Barbed reposition pharyngoplasty (BRP)		Soft palate and pharyngeal wall suspension using barbed sutures without knots	Extrusion rate ~18.4%; no significant impact on QoL or polygraphy outcomes	Minimally invasive; technically simple; cost-effective	Suture extrusion complication; long-term durability uncertain	Ideal for patients with lateral wall collapse and a poor response expected from traditional UPP
Laser-assisted uvulopalatoplasty (LAUP)		Laser resection of the uvula and palatal tissue to widen the oropharyngeal airway	~50% achieve AHI < 10	Outpatient procedure; minimally invasive	High complication rates (dysphagia, VPI, uvular necrosis)	Ideal for patients seeking a less invasive approach, with palatal and lateral wall collapse
Maxillomandibular advancement (MMA)		Advancement of the maxilla and mandible to enlarge the retropalatal and retrolingual airway space	AHI 63.9 → 9.5; success ~86%; cure (AHI < 5): 43–47%	Highest surgical success rate; multilevel airway expansion; durable effect	-Highly invasive -Side effects: facial edema and neurosensory complications	Ideal for patients with craniofacial abnormalities, retrognathia, multilevel obstruction, or elevated upper-airway collapsibility (high Pcrit)
Genioglossus advancement ± hyoid suspension		Anterior repositioning of tongue musculature and stabilization of the hypopharynx	Modest AHI reduction -Inferior to MMA	Targeted hypopharyngeal intervention is often adjunctive	Limited efficacy as a standalone therapy	Ideal for tongue-base collapse or hypopharyngeal obstruction identified on DISE
Inferior sagittal mandibular osteotomy (tongue base procedures)		Structural anterior repositioning of the tongue base via mandibular manipulation	Moderate improvement in airflow	Useful in multilevel surgery protocols	Insufficient as monotherapy	Ideal for patients with significant tongue-base obstruction as part of a multilevel surgical strategy

**Table 3:** Comparison of PAP modalities for the management of sleep-related breathing disorders.

Modality	Mechanism/Key Feature	Clinical Effect/Outcomes	Advantages	Limitations/Considerations
Continuous Positive Airway Pressure (CPAP)	Delivers fixed positive airway pressure to maintain upper airway patency during sleep	-Reduced AHI and daytime sleepiness -Improvement in QoL	Widely available and well-established efficacy	-Requires titration in-lab in traditional systems -Adherence limited by mask discomfort and pressure intolerance
Auto-adjusting Positive Airway Pressure (APAP)	Automatically adjusts pressure on a breath-by-breath basis in response to airflow dynamics	Comparable efficacy to CPAP with no significant difference in AHI reduction	Does not require a titration study like CPAP → increased compliance	Not appropriate for CSA or hypoventilation syndromes
Bilevel Positive Airway Pressure (BIPAP)	Delivers constant pressure throughout the respiratory cycle via inspiratory PAP (IPAP) and expiratory PAP (EPAP) to reduce the work of breathing	-Similar improvements to CPAP in AHI, sleepiness, and QoL -Beneficial in patients requiring higher pressures or ventilatory support	-Similar improvements to CPAP in AHI, sleepiness, and QoL -Beneficial in patients requiring higher pressures or ventilatory support	More complex titration; no clear superiority over CPAP for routine OSA
Volume Assisted Pressure Support (VAPS)	Automatically adjusts IPAP to achieve a target tidal volume or minute ventilation	Improves ventilation and CO_2_ clearance in hypoventilation syndromes (e.g., OHS, neuromuscular disease)	-Provides guaranteed ventilation -Adaptive support for hypoventilation	-Not indicated for uncomplicated OSA -Limited evidence in general OSA populations
Adaptive Sero-ventilation (ASV)	Provides breath-by-breath adjustment of inspiratory support based on detected ventilation to stabilize breathing patterns	-Highly effective in CSA and treatment-emergent CSA -Reduces residual AHI and improves sleep quality	-Superior control of complex sleep-disordered breathing -Adaptive real-time ventilation targeting	-Contraindicated in selected heart failure populations with reduced ejection fraction -Limited use outside CSA phenotypes

## Data Availability

No new data were created or analyzed in this study.

## Abbreviations

The following abbreviations are used in this manuscript:

SRBD	Sleep-Related Breathing Disorders
OSA	Obstructive Sleep Apnea
CSA	Central Sleep Apnea
ROS	Reactive Oxygen Species
PAP	Positive Airway Pressure
EEG	Electroencephalogram
EOG	Electrooculogram
EKG	Electrocardiogram
AHI	Apnea–Hypopnea Index
PAT	Peripheral Arterial Tonometry
EMG	Electromyography
OTC	Optical Coherence Tomography
BMI	Body Mass Index
AASM	American Academy of Sleep Medicine
CPAP	Continuous Positive Airway Pressure
BiPAP	Bilevel Positive Airway Pressure
APAP	Autotitrating Positive Airway Pressure
REM	Rapid Eye Movement
MAD	Mandibular Advancement Device
TMJ	Temporomandibular Joint
DISE	Drug-Induced Sleep Endoscopy
UPPP	Uvulopalatopharyngoplasty
ESP	Expansion Sphincter Pharyngoplasty
BRP	Barbed Reposition Pharyngoplasty
LAUP	Laser-Assisted Uvulopalatoplasty
MMA	Maxillomandibular Advancement
NREM	Non-Rapid Eye Movement
GAHM	Genioglossus Advancement and Hyoid Myotomy
RFA	Radiofrequency Ablation
TORS	Transoral Robotic Surgery
VSP	Virtual Surgical Planning
3D	Three-Dimensional
HNS	Hypoglossal Nerve Stimulation
IPAP	Inspiratory Positive Airway Pressure
EPAP	Expiratory Positive Airway Pressure
VAPS	Volume-Assisted Pressure Support
BIPAP S/T	Bilevel Positive Airway Pressure with a backup rate
ASV	Adaptive Servo-Ventilation
HFrEF	Heart Failure with Reduced Ejection Fraction
PPAR-ɣ	Peroxisome Proliferator-Activated Receptor Gamma
CIH	Chronic Intermittent Hypoxia
DEG	Differentially Expressed Gene
NO	Nitric Oxide
PTSD	Post-Traumatic Stress Disorder
AI	Artificial Intelligence
PCDM	Patient Centered Decision Making
PCSR	Patient Centered Sleep Study Report
